# *Staphylococcus aureus* accessory gene regulator quorum-sensing system inhibits keratinocyte lipid enzymes and delays wound repair

**DOI:** 10.1172/JCI190411

**Published:** 2025-10-15

**Authors:** Michelle D. Bagood, Jelena Marjanovic, Nina Jiang, Hung Chan, Tatsuya Dokoshi, Kellen J. Cavagnero, Fengwu Li, Andrea Roso-Mares, Samia Almoughrabie, Edward Liu, Irena Pastar, Marjana Tomic-Canic, Alexander R. Horswill, Richard L. Gallo

**Affiliations:** 1 Department of Dermatology, University of California San Diego, La Jolla, California, USA.; 2 Wound Healing and Regenerative Medicine Research Program, Dr. Phillip Frost Department of Dermatology and Cutaneous Surgery, University of Miami Miller School of Medicine, Miami, Florida, USA.; 3 Department of Immunology and Microbiology, University of Colorado Anschutz Medical Campus, Aurora, Colorado, USA.

**Keywords:** Dermatology, Infectious disease, Bacterial infections, Innate immunity, Skin

## Abstract

Mechanisms responsible for delayed wound repair are poorly understood despite the common impact of this disorder on health. To study how *Staphylococcus aureus* disrupts healing, mouse and human wound repair models were evaluated after exposure to *S*. *aureus* or commensal *Staphylococcus*. Quorum sensing by *S*. *aureus*, but not *S*. *hominis*, delayed repair and inhibited the expression of genes responsible for lipid metabolism in keratinocytes. *S*. *aureus* with inactive accessory gene regulator (agr) did not delay healing, and the inhibition of lipid metabolism was recapitulated in vitro by synthetic phenol soluble modulin α1 (psmα1) and psmα4, genes that are under agr control. However, *S*. *aureus* strains with single deletion of *psmA*, *psmB*, alpha-hemolysin (*hla*), or *hld* gene continued to delay repair, suggesting that *S*. *aureus* used multiple agr-dependent virulence factors to disrupt healing. These observations provide insight into mechanisms for delayed wound healing, identify quorum sensing as a critical event, and highlight the role of lipid biosynthesis in wound reepithelialization.

## Abstract 2

<span>Staphylococcus aureus delays wound healing by triggering quorum sensing and disrupting the expression of key genes involved in lipid synthesis and barrier formation.</span>

## Introduction

Methicillin-resistant *Staphylococcus aureus* (MRSA) is one of the most common infectious agents associated with delayed wound healing (1). One important mechanism by which MRSA establishes itself in wounds is by producing biofilms (2, 3). This can frequently complicate diabetic foot ulcers (DFUs) and is associated with substantial morbidity, with approximately 1 in 6 DFUs resulting in lower extremity amputation. Analysis of mechanisms through which MRSA inhibits healing has uncovered multiple potential modes of action (4–6). In particular, the presence of multiple antibiotic-resistance genes, resulting in increased survival of bacteria in the wound, is linked to poor diabetic wound healing (7). Therefore, alternative nonantibiotic approaches, such as targeting bacterial virulence, are urgently needed.

A critical factor in predicting a negative effect of *S*. *aureus* in wounds is the absolute abundance of the organism at the site, with prior clinical studies suggesting that infection and delayed repair occur if *S*. *aureus* exceeds a threshold of 10^5^ CFU per gram of tissue (8). Given that increased bacterial density results in the activation of quorum-sensing systems, like the accessory gene regulator (agr) system, these observations suggest that the quorum-sensing process may be important. Supporting this, several studies on agr quorum sensing–controlled virulent products, such as alpha toxin and Panton-Valentine leucocidin, have suggested these products may impair healing and set the stage for chronicity (9–11).

In contrast to the well-established capacity of *S*. *aureus* to delay wound healing, much less is known about the effects of other species of *Staphylococcus* that are abundant on the skin yet not frequently reported to induce infections. Indeed, some studies suggest that the presence of commensal bacteria or their bioactive compounds may improve healing (12–14). Pathways invoked for the benefits of bacteria in wounds include induction of tissue hypoxia-activated HIF-1α signaling, which can increase glutamine metabolism in keratinocytes, and the production of IL-1β (15). In other murine skin injury models, commensal bacteria have been shown to activate neutrophils to produce CXCL10, which recruits and activates plasmacytoid DCs to produce type 1 IFNs that accelerate wound healing via T cell–dependent and –independent mechanisms (16). Overall, the role of microbes in wound repair remains poorly understood.

The complex process of wound repair involves acute inflammation, differentiation, cell migration, and regeneration. Each step provides an opportunity for microbes to influence the process. For example, in a wound-induced hair neogenesis model, *S*. *aureus* infection resulted in regeneration by providing a persistent inflammatory signal (17). Effective healing, however, is dependent on reestablishing the epithelial barrier, a structure that is dependent in part on the capacity of epidermal keratinocytes to control the synthesis of lipids. Keratinocytes in the stratum corneum must form a neutral lipid-enriched extracellular matrix mainly composed of ceramides, cholesterol, and free fatty acids to re-form the barrier (18). Furthermore, the spatial distribution and relative abundance of lipids correlate with stages of epithelialization in human skin (19). The influence of microbes on lipid synthesis in wounds has not been extensively studied despite the important role that microbes play in lipid synthesis by keratinocytes (20). These examples of microbiota-host interactions show the nuanced relationship between microbes and wound repair and highlight the importance of further, more detailed study of these processes.

In this study, we sought to better understand the mechanisms of *S*. *aureus*–mediated inhibition of wound healing by comparison to a human skin commensal species of *Staphylococcu*s and by evaluation of the effects of different bacterial loads. We utilized bulk, spatial, and cell-specific RNA-Seq technology and interrogated the host cellular response to wound infection using a variety of human and murine models. We then combined this approach with bacterial mutants to pinpoint specific *S*. *aureus*–derived products responsible for delayed repair. We showed that pathogenic *S*. *aureus*, not commensal *S*. *hominis*, uniquely impaired healing, and this delay was dependent on the activation of the *S*. *aureus* agr quorum-sensing system. This infection resulted in altered keratinocyte lipid metabolism across models, enhancing our understanding of the role of microbes during wound repair.

## Results

### S. aureus, not S. hominis, inhibits repair and alters the expression of lipid synthesis genes.

**Figure 1:**
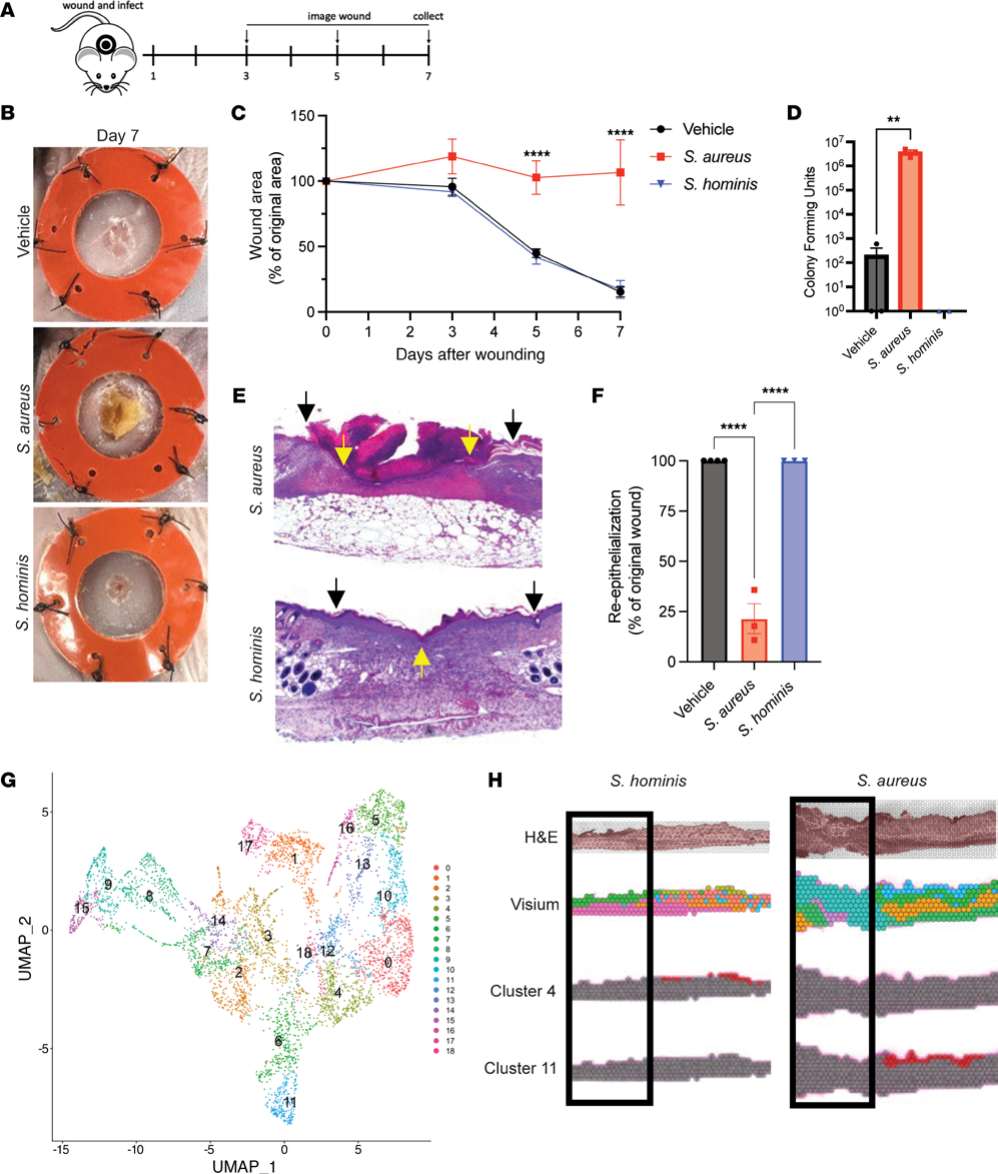
*S*. *aureus,* not *S*. *hominis*, delays cutaneous wound healing. (**A**) Schema of mouse infected wound model. (**B**) Representative images of murine wounds on day 7 after inoculation with vehicle, *S*. *aureus*, or *S*. *hominis* (1 × 10^7^ CFU/wound in 10 μL gel). (**C**) Quantification of wound closure based on analysis of wound images on days after wounding 0, 3, 5, and 7. (**D**) Quantification of live bacteria cultured from wounds on day 7. (**E**) Representative H&E-stained histology images of *S*. *aureus*– and *S*. *hominis*–inoculated wounds on day 7 (black arrows: original wound edge; yellow arrows: end of neo-epithelial tongue). (**F**) Quantification of reepithelialization of vehicle-, *S*. *aureus*–, and *S*. *hominis*–inoculated wounds on day 7 based on analysis of H&E histology images. (**G**) Unbiased clustering of Visium spatial RNA-Seq data from intact and wounded murine skin 1 day after treatment with vehicle, *S*. *aureus*, or *S*. *hominis*. UMAP, uniform manifold approximation and projection. (**H**) Representative images of H&E, spatial location of all Visium clusters, and either cluster 4 or 11 highlighted (wound area highlighted by box). Experiments were performed at least twice unless otherwise indicated. One-way ANOVA followed by Dunnett’s multiple-comparison adjustment for more than 2 groups (**C**, **D**, and **F**). Data represent mean ± SEM. **P < 0.01, ****P < 0.0001.

To establish a reliable model system and address contradictory evidence regarding the effect of bacteria on wound closure (4, 15–17), we first tested a splinted, full-thickness skin wound model in mice. Wounds were inoculated with equal amounts (1 × 10^7^ CFU) of *S*. *hominis* (a skin commensal strain of Staphylococcus) or *S*. *aureus* (the most common bacteria found in nonhealing wounds) or vehicle. Wounds were imaged on days 0, 1, 3, 5, and 7 after wounding to track wound closure, and wounds were collected on day 7 for histology to measure reepithelialization (Figure 1A). Based on wound images (Figure 1B), *S*. *hominis*–inoculated wounds closed and reepithelialized similar to vehicle-treated wounds with no live CFUs of *S*. *hominis* present on day 7. However, *S*. *aureus*–infected wounds showed delayed repair and abundant *S*. *aureus* CFUs in the wound on day 7 (Figure 1, C–F). These results show that at similar initial inoculates, *S*. *hominis* did not inhibit the healing of skin wounds in mice, whereas *S*. *aureus* significantly impaired repair.

**Figure 2:**
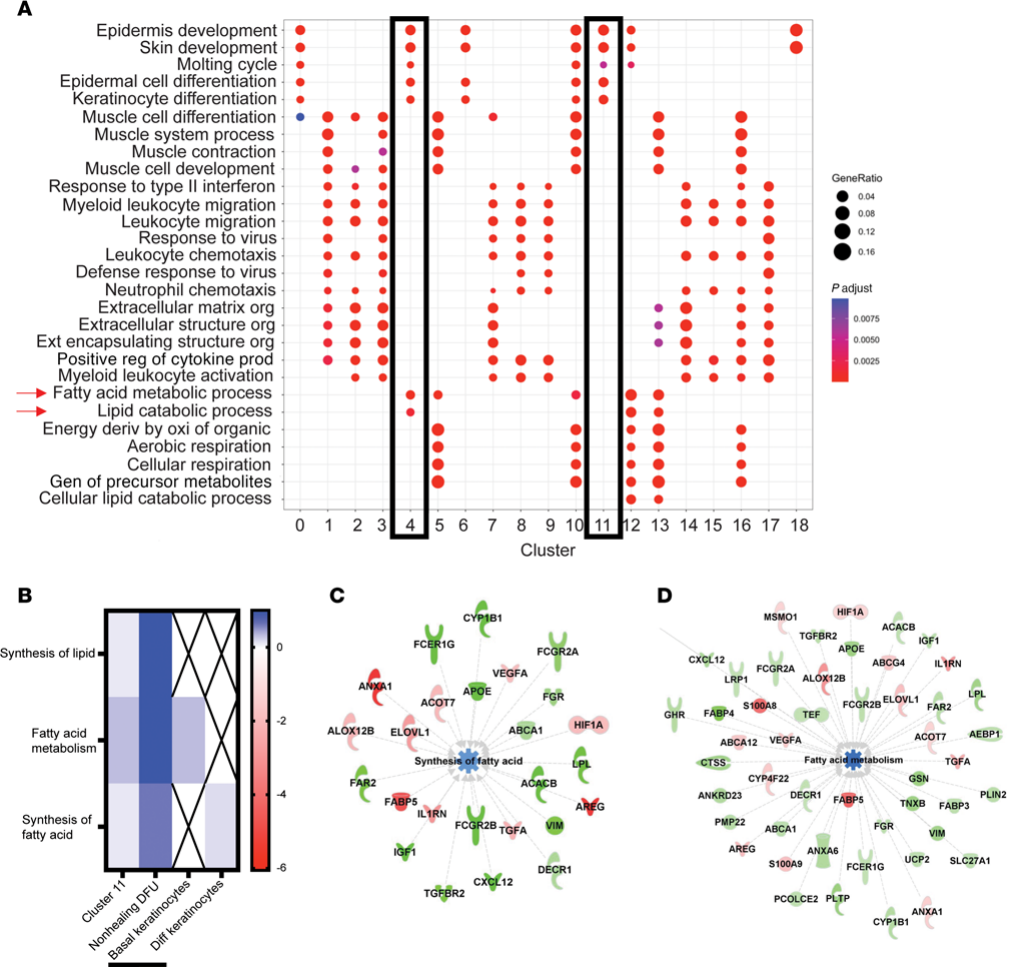
Transcriptional analysis of mouse and human wounds infected by *S*. *aureus*. (**A**) Pathway analysis of Visium clusters with clusters 4 and 11 highlighted. (**B**) Heatmap of fatty acid pathways comparing mouse cluster 11, nonhealing human diabetic foot ulcer (DFU) RNA-Seq data, and deconvoluted keratinocyte subpopulations from DFU RNA-Seq data. (**C**) Ingenuity pathway analysis (IPA) overlapping targets from cluster 11 and nonhealing human DFU transcriptomic profiles for synthesis of fatty acid pathway. (**D**) IPA identified overlapping targets from cluster 11 and nonhealing DFU datasets for fatty acid metabolism. Experiments were performed at least twice unless otherwise indicated. One-way ANOVA followed by Dunnett’s multiple-comparison adjustment for more than 2 groups. Data represent mean ± SEM.

We next evaluated gene expression in wound tissue by spatial transcriptomics. Nineteen cell clusters were defined by unbiased clustering of the spatial Visium RNA-Seq data obtained from wounds 24 hours after injury and exposure to either *S*. *aureus* or *S*. *hominis* (Figure 1G). Clusters 0, 4, 6, 10, 11, 12, and 18 were identified as likely to be regions occupied primarily by keratinocytes based on top 3 genes expressed, morphological location, and functional pathway analysis (Supplemental Figure 1A, Figure 1H, and Figure 2A; supplemental material available online with this article; https://doi.org/10.1172/JCI190411DS1). Spatial visualization showed cluster 4 was present only in the epidermis of the *S*. *hominis*–exposed wound, whereas wounds exposed to *S*. *aureus* lost expression of genes in cluster 4 and were enriched with genes present in cluster 11 of the peri-wound epithelium (Figure 1H). Pathway analysis showed similar expression of genes related to epidermal development and differentiation between these clusters, but genes involved in the fatty acid metabolic processes and lipid catabolic process found in cluster 4 were not detected in the *S*. *aureus*–infected wound (Figure 2A). Similarly, differential expression analysis of clusters 4 and 11 showed cluster 4 had higher expression of multiple genes involved in cellular metabolism, and the *S*. *aureus*–associated cluster 11 had a prevalence of host defense genes (Supplemental Figure 1B). These data show that wounds inoculated with *S*. *hominis* maintained metabolic processes that are associated with physiological healing, and wounded epidermis exposed to *S*. *aureus* was marked with the suppression of genes involved in lipid metabolism.

To investigate the potential clinical relevance of dysregulated fatty acid metabolism and synthesis observed in mouse wounds, we next studied the transcriptional response of human wounds. Comparative genomics using Ingenuity Pathway Analysis (IPA; QIAGEN) was done on bulk RNA-Seq data from human nonhealing DFUs. Comparison between mouse cluster 11 genes and the transcripts detected in human DFUs revealed that the nonhealing DFUs had similar dysregulation of genes involved in lipid and fatty acid metabolism (Figure 2B). Further, the deconvoluted human DFU RNA-Seq data showed the basal keratinocyte subpopulation also downregulated fatty acid metabolism genes and differentiated keratinocytes downregulated synthesis of fatty acid genes (Figure 2B). A subset of 25 genes involved in fatty acid synthesis and a subset of 48 genes involved in fatty acid metabolism showed the same directionality of expression between nonhealing human DFUs and cluster 11 from mouse wounds (Figure 2, C and D), demonstrating suppression of both fatty acid metabolism and synthesis in both models (Figure 2, C and D, and Supplemental Data 1–4). Taken together, these observations suggest a relationship between delayed healing and deregulation of keratinocyte lipid and fatty acid metabolism.

### S. aureus inhibits the expression of lipid synthesis genes by keratinocytes.

**Figure 3:**
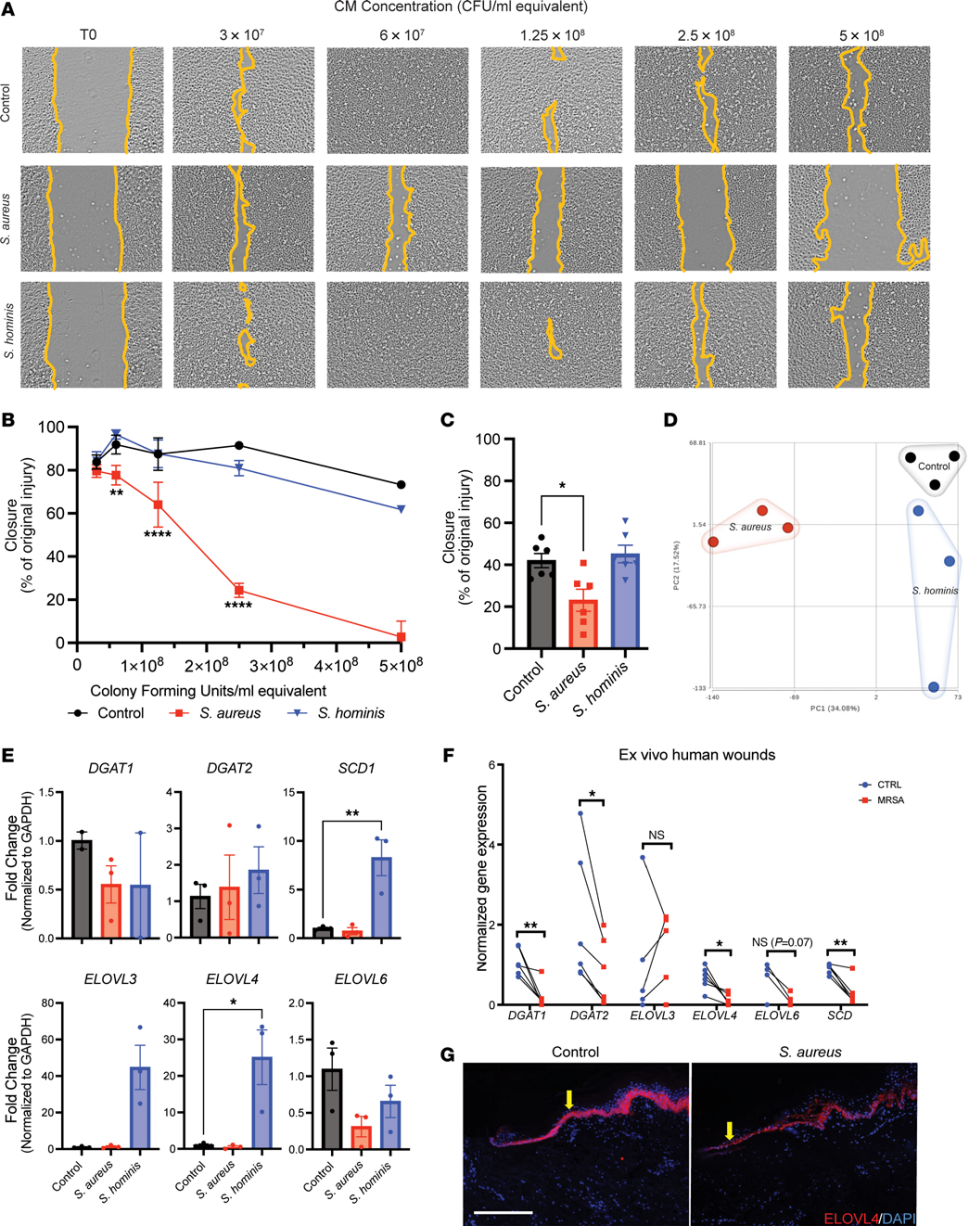
*S*. *aureus* inhibits keratinocyte function and expression of genes involved in lipid biosynthesis. (**A**) Representative images of HaCaT cells scratch-wound in vitro assay at time 0 and after 24 hours treatment with increasing doses of sterile conditioned media (CM) from *S*. *aureus* and *S*. *hominis*. CM dose is indicated as equivalent to bacterial CFU/mL. (**B**) Quantification of HaCaT cells scratch-wound closure in vitro treated with increasing doses of CM for 24 hours. (**C**) Quantification of in vitro neonatal human epidermal keratinocytes (NHEKs) scratch-wound closure 24 hours after treatment with CMs (3 × 10^7^ CFU/mL equivalent) or media-only control. (**D**) Principal component analysis (PCA) plot of RNA sequencing results from scratch-wounded NHEKs treated for 24 hours with CM (3 × 10^7^ CFU/mL equivalent) from *S*. *aureus*, S. *hominis*, or media-only control. (**E**) Lipid synthesis gene expression of scratch-wounded NHEKs in vitro treated for 24 hours with CM from *S*. *aureus*, *S*. *hominis*, or media-only control. (**F**) Lipid synthesis gene expression in control or *S*. *aureus*–infected (MRSA) ex vivo human wounds. (**G**) Representative immunofluorescence images of ex vivo human nondiabetic skin with and without addition of *S*. *aureus* and stained for ELOVL4 (scale bar: 300 μm). One-way ANOVA followed by Bonferroni’s multiple-comparison adjustment for more than 2 groups (**B**, **C**, and **E**), multiple Student’s *t* test for comparison with control group (**F**). Experiments were performed at least twice. Data represent mean ± SEM. *P < 0.05, **P < 0.01, ****P < 0.0001.

Based on the spatial transcriptomic findings that highlighted a damaging effect of *S*. *aureus* on keratinocytes at the wound edge, and the correlation of this data with bulk analysis of human nonhealing DFUs, we next focused our analysis on cultured human keratinocytes to further define human keratinocyte genes that are directly influenced by the products of *S*. *aureus*. To reflect exposure to bacterial products at a range of concentrations, immortalized keratinocytes (HaCaT) were exposed to increasing volumes of sterile-filtered conditioned media (CM) from cultures of *S*. *aureus* and *S*. *hominis*. Similar volumes of unused bacterial culture media were added to separate cell cultures, serving as a control. Cytotoxicity in HaCaT cultures based on LDH release occurred at only the highest concentration of *S*. *aureus* CM and was not used for further analysis (Supplemental Figure 2A). HaCaT migration was inhibited by CM at concentrations equivalent to 6 × 10^7^ CFU/mL of *S*. *aureus* (Figure 3, A and B). In contrast, *S*. *hominis* CM at these densities and above did not inhibit keratinocyte migration (Figure 3, A and B). Similarly, all but the lowest concentration of *S*. *aureus* CM inhibited keratinocyte proliferation, whereas *S*. *hominis* CM showed no detectable difference compared with the control (Supplemental Figure 2, B and C). This differential response to *S*. *aureus* CM compared with *S*. *hominis* CM also occurred in primary neonatal human keratinocytes (NHEKs), although the primary human cell cultures were more susceptible than HaCaT to *S*. *aureus* CM (Supplemental Figure S2, D and E). With exposure to 3 × 10^7^ CFU/mL equivalent concentration of *S*. *aureus* CM, the relative response of NHEKs was similar to HaCaT, demonstrating *S*. *aureus* inhibited keratinocyte migration and proliferation more than *S*. *hominis* in both in vitro models (Figure 3C and Supplemental Figure 2, F and G).

Next, NHEKs were evaluated by bulk RNA-Seq after exposure to *S*. *aureus*, *S*. *hominis*, or unspent bacterial culture media control. Principal component analysis revealed NHEKs exposed to *S*. *hominis* products clustered close to control keratinocytes, suggesting these groups have similar transcriptomes, while the presence of *S*. *aureus* products resulted in a shift of the transcriptome and a distinct cluster away from the control (Figure 3D). This was supported by a heatmap of differential gene expression of *S*. *aureus* and *S*. *hominis* CM treatment showing broad differential expression of genes influenced by *S*. *aureus* compared with *S*. *hominis* (Supplemental Figure 3A). Gene ontology and differential gene expression analysis revealed NHEKs exposed to *S*. *aureus* CM treatment upregulated host defense and cell-cell adhesion pathways, and *S*. *hominis* CM-treated NHEKs increased α-amino acid metabolism and apoptosis pathways (Supplemental Figure 3B). Differentially expressed gene analysis was validated with qPCR for several genes in these pathways (Supplemental Figure 3C). Comparison of the murine samples, in vitro keratinocytes, and nonhealing DFU samples by IPA to bulk RNA-Seq results from acute, normal healing human skin wounds demonstrated consistent suppressed gene pathways in *S*. *aureus*–infected murine wounds, keratinocytes exposed to *S*. *aureus* products, and human DFUs with delayed repair (Supplemental Figure 3D).

**Figure 4:**
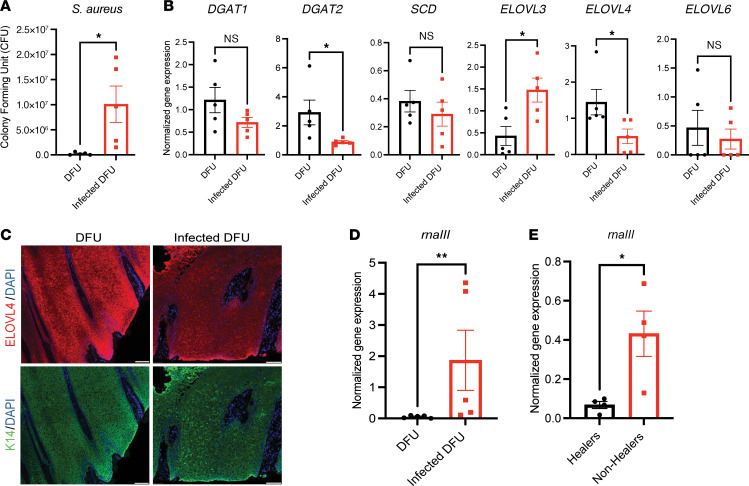
Human wounds demonstrate decreased lipid enzymes in the presence of *S*. *aureus*. (**A**) *S*. *aureus* quantification in human diabetic foot ulcers (DFUs) distinguishes those with clinical infection. (**B**) qPCR of lipid biosynthesis enzyme genes in infected and noninfected DFUs. (**C**) Representative images of DFU and infected DFU immunofluorescence stained for ELOVL4 and K14 (scale bar: 100 μm). (**D**) qPCR quantification of *S*. *aureus* *rnaIII* in infected and noninfected DFUs. (**E**) qPCR quantification of *S*. *aureus* *rnaIII* in healing and nonhealing DFUs. Experiments were performed at least twice. Student’s *t* test for comparison of 2 groups (**A**, **B**, **D**, and **E**). Data represent mean ± SEM. *P < 0.05, **P < 0.01.

Reverse transcription quantitative real-time PCR (RT-qPCR) analysis was performed to further assess the expression of critical enzymes for lipid biosynthesis that are present in keratinocytes (Supplemental Figure 3E). Commensal *S*. *hominis*, but not *S*. *aureus*, induced expression of *SCD*, *ELOVL3*, and *ELOVL4* in cultured keratinocytes (Figure 3E). Human skin biopsies infected ex vivo with *S*. *aureus* also showed suppression of genes involved in triglyceride and fatty acid synthesis compared with the control (Figure 3F). Immunostaining for ELOVL4 showed decreased protein expression in human skin after infection with *S*. *aureus* (Figure 3G). Furthermore, utilizing *S*. *aureus*–specific *nucA* amplification, we confirmed that clinically infected DFU samples had more *S*. *aureus* compared with noninfected DFUs (Figure 4A), and a decrease of *DGAT2* and *ELOVL4* gene and protein expression occurred in *S*. *aureus*–infected DFUs compared with uninfected DFUs (Figure 4, B and C). These results further support the observation that *S*. *aureus* in murine and human wounds affects the expression of genes involved in lipid synthesis and these responses correlate with delayed repair.

### S. aureus agr activity is required for the inhibition of wound healing.

To better understand how *S*. *aureus* influences repair response, we next measured the expression of *rnaIII*, a key *S*. *aureus* regulator in agr quorum sensing. Consistent with the activation of quorum sensing by higher bacterial density in the infected wounds, *rnaIII* was induced in infected human DFUs compared with uninfected DFUs (Figure 4D). This pattern of induction was also linked to clinical outcomes of healing as *rnaIII* gene induction was found in nonhealing DFUs when compared with healing DFUs (Figure 4E).

**Figure 5:**
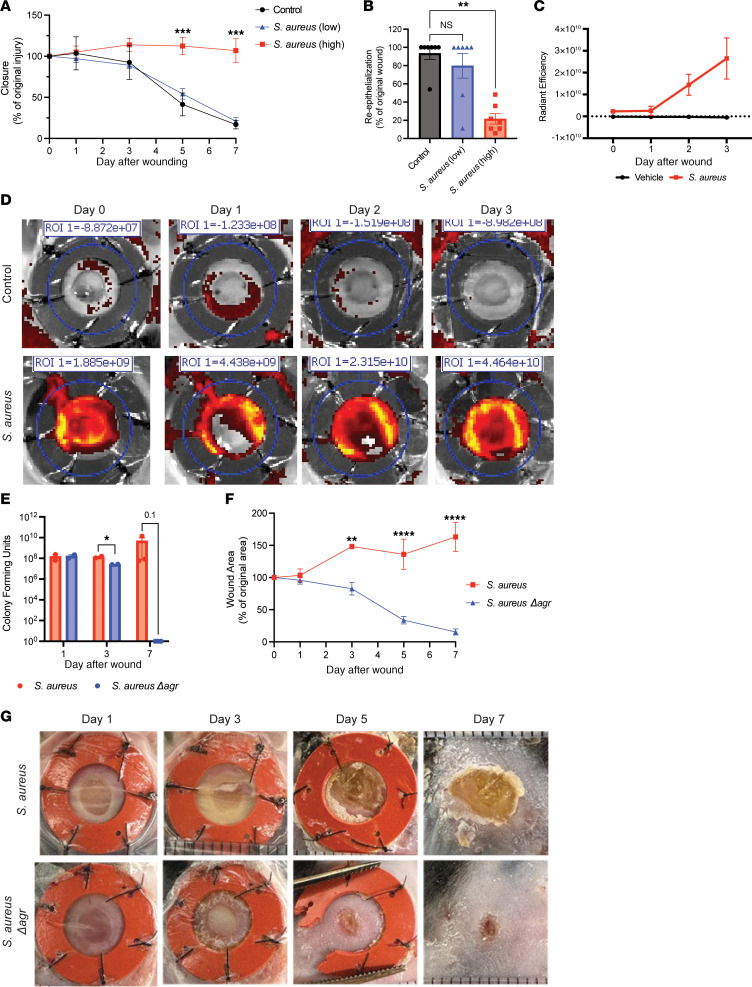
Activation of agr quorum sensing in *S*. *aureus* inhibits wound healing. (**A**) Quantification of murine wound closure over time (days 0, 1, 3, 5, 7) inoculated with low (1 × 10^4^ CFU/wound in 10 μL gel) or high dose (1 × 10^7^ CFU/wound in 10 μL gel) of *S*. *aureus* or vehicle control (10 μL). (**B**) Quantification of reepithelialization based on H&E histology image analysis of wounds on day 7 after exposure to vehicle control or low or high dose of *S*. *aureus*. (**C**) Quantification of IVIS images confirming activation of agr by *S*. *aureus* in vivo. (**D**) IVIS imaging of control and *S*. *aureus* agr reporter strain infected mouse wounds. ROI, region of interest. (**E**) Live bacteria cultured from infected murine wounds on days 1, 3, and 7. (**F**) Quantification of murine wound closure after infection with *S*. *aureus* or *S*. *aureus*Δ*agr* (days 0, 1, 3, 5, and 7). (**G**) Representative images of WT *S*. *aureus* and agr mutant (*S*. *aureusΔagr*) infected wounds (days 1, 3, 5, and 7). Experiments were performed at least twice. Student’s *t* test for comparison of 2 groups (**C**, **E**, and **F**), 1-way ANOVA followed by Bonferroni’s multiple-comparison adjustment for more than 2 groups (**A**, **B**, and **D**). Data represent mean ± SEM. *P < 0.05, **P < 0.01, ***P < 0.001, ****P < 0.0001.

Next, to test whether the activation of the *S*. *aureus* agr system was necessary for delayed wound repair, murine wounds were topically inoculated with low (10^4^ CFU/wound) or high (10^7^ CFU/wound) doses of *S*. *aureus*. The low dose of *S*. *aureus* was controlled by the host and did not inhibit the closure or reepithelialization of wounds, whereas the high dose impaired both (Supplemental Figure 4A and Figure 5, A and B). The high density of *S*. *aureus* resulted in activation of the agr quorum-sensing system at day 1 as detected by an *S*. *aureus* agr reporter strain (Figure 5, C and D). At this high density of *S*. *aureus* (10^7^ CFU/wound), a mutant strain of *S*. *aureus* lacking the agr system (*Sa*Δ*agr*) showed decreased purulence, lower bacterial burden, and healed normally by day 7 (Figure 5, E–G, and Supplemental Figure 4, B and C). *Sa*Δ*agr* grew similarly to the parental WT strain in permissive bacterial media (Supplemental Figure 4D). Thus, activation of *S*. *aureus* agr is essential for this pathogen to delay wound repair in mice.

**Figure 6:**
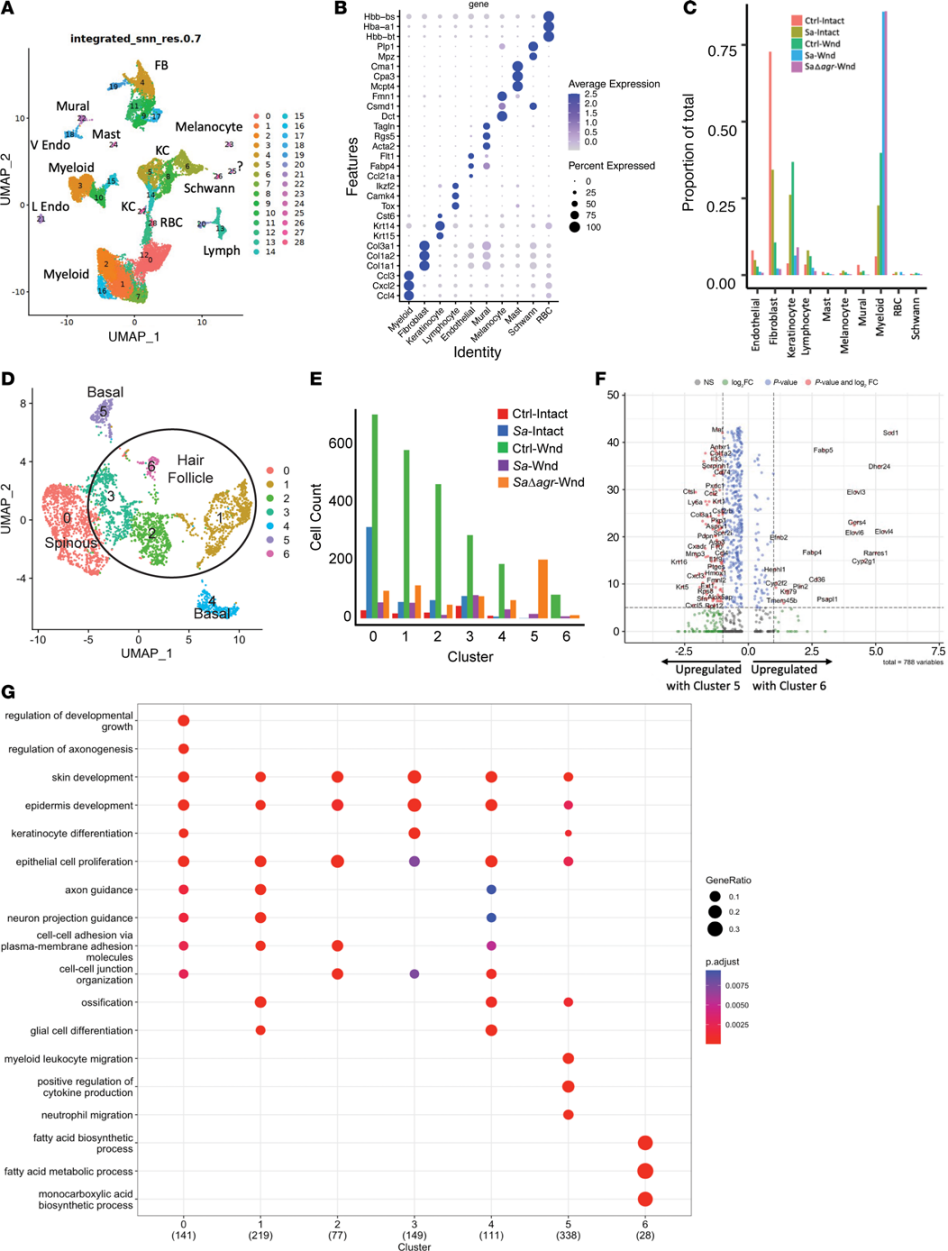
Single-cell transcriptomics of murine wounds inoculated with *S*. *aureus* identify keratinocytes as a target of agr activation. (**A**) Unbiased clustering of single-cell RNA-Seq data from intact murine skin and skin 24 hours after wounding and inoculation with *S*. *aureus*, *S*. *aureusΔagr*, or vehicle control. (**B**) Top 3 overrepresented markers by cell type. (**C**) Quantification of proportions of each cell type based on treatment group. (**D**) Unbiased clustering of keratinocyte subsets. (**E**) Counts of each keratinocyte cluster by treatment. (**F**) Volcano plot of differentially expressed genes identified in keratinocyte clusters 5 and 6 by DESeq analysis. (**G**) Gene Ontology overrepresentation analysis of the 7 keratinocyte clusters. Experiments were performed twice.

Transcriptional analysis of mouse wounds infected with *Sa*Δ*agr* compared with WT *S*. *aureus*, as well as intact skin treated with vehicle or WT *S*. *aureus*, was then performed by single-cell RNA-Seq of peri-wound tissue 24 hours after wounding and bacterial inoculation. Unbiased cell clustering of the pooled single-cell RNA-Seq data defined 29 clusters (Figure 6A), and top 3 marker expression enabled identification of cluster cell types (Figure 6B). Comparison of the proportion of various cell types between experimental groups found that the proportion of myeloid cells in the total population increased with topical *S*. *aureus* exposure over topical vehicle control (Figure 6C). Wounding alone further increased the myeloid population, but the greatest proportion of myeloid cells was found in the *S*. *aureus*– and *Sa*Δ*agr*-inoculated wound tissue (Figure 6C). The proportion of keratinocytes remained similar in the peri-wound tissue treated with either *S*. *aureus* or *Sa*Δ*agr* (Figure 6C). Taken together, we see that *S*. *aureus* required the agr quorum-sensing system to delay wound repair but did not result in a major difference in keratinocytes recovered from the epidermis surrounding the wound. Thus, these populations of keratinocytes were suitable for further comparison.

Unbiased clustering of keratinocytes defined 7 subclusters of keratinocytes from normal, infected, and wounded skin (Figure 6D). Assessing top marker expression with previous peri-wound single-cell analysis (21), we defined cluster 0 as spinous keratinocytes, clusters 1 to 3 and 6 as keratinocytes associated with the hair follicle, and clusters 4 and 5 as basal keratinocytes (Supplemental Figure 4E and Figure 6D). Clusters 5 and 6 were overrepresented in the wound samples (Figure 6E). Noninfected wounds had the highest cell counts in all keratinocyte subpopulations except cluster 5, which was most abundant in the *Sa*Δ*agr*-inoculated wound (Figure 6E). *S*. *aureus* infection reduced all cluster counts, and *Sa*Δ*agr* partially restored counts compared with infection with WT *S*. *aureus* in most clusters (Figure 6E).

Differentially expressed genes and functional pathway analysis showed an increase in the prevalence of host defense genes in cluster 5 and increased genes for enzymes important for fatty acid biosynthesis and metabolic processes in cluster 6 (control wound) (Figure 6, F and G). These data showed that *S*. *aureus* infection inhibited expression of genes for lipid metabolic functions and were consistent with results from spatial sequencing and qPCR of ex vivo human wounds.

### S. aureus agr activity is required to inhibit keratinocyte function.

**Figure 7:**
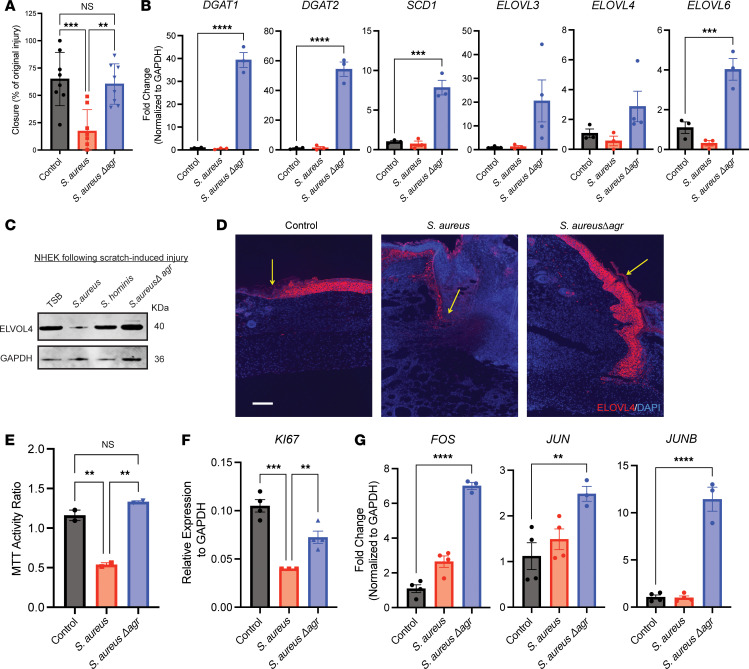
*S*. *aureus* agr system influences keratinocyte function and gene expression. (**A**) Quantification of in vitro NHEK scratch-wound closure 24 hours after treatment with *S*. *aureus* or *S*. *aureusΔagr* CM (3 × 10^7^ CFU/mL equivalent), or media control. (**B**) qPCR for lipid metabolic/synthesis enzyme genes of scratch-wounded in vitro NHEKs treated for 24 hours with *S*. *aureus* or *S*. *aureusΔagr* CM (3 × 10^7^ CFU/mL equivalent), or media control (control and *S*. *aureus* groups shown previously in Figure 3E). (**C**) Protein immunoblot of ELOVL4 and GAPDH protein extracted from scratch-injured NHEKs exposed to *S*. *aureus*, *S*. *hominis*, or *S*. *aureusΔagr* CM (3 × 10^7^ CFU/mL equivalent), or media control for 24 hours. (**D**) Representative images of murine wounds infected with vehicle, *S*. *aureus*, or *S*. *aureusΔagr* immunofluorescence-stained for ELOVL4 (scale bar: 300 μm). (**E**) MTT proliferation assay of in vitro NHEKs after 24 hours of treatment with *S*. *aureus* or *S*. *aureusΔagr* CM (3 × 10^7^ CFU/mL equivalent), or media control. (**F**) *KI67* gene expression of proliferating NHEKs after 24 hours of treatment with *S*. *aureus* or *S*. *aureusΔagr* CM (3 × 10^7^ CFU/mL equivalent), or media control. (**G**) Expression of cell cycle genes by in vitro NHEKs after 24 hours of treatment with *S*. *aureus* or *S*. *aureusΔagr* CM (3 × 10^7^ CFU/mL equivalent), or media control. Experiments were performed at least twice. One-way ANOVA followed by Bonferroni’s multiple-comparison adjustment for more than 2 groups. Data represent mean ± SEM. **P < 0.01, ***P < 0.001, ****P < 0.0001.

Based on the requirement for *S*. *aureus* agr quorum sensing to delay repair and influence some transcriptional responses in keratinocytes, we hypothesized that keratinocyte function would be affected by the soluble products of the agr system. To test this, we investigated the effects of the CM from *S*. *aureus* and *Sa*Δ*agr* on NHEKs in culture. Deletion of agr activity from *S*. *aureus* enabled normal NHEK migration (Figure 7A), reduced CM-dependent cytotoxicity as measured by LDH release (Supplemental Figure 5A), and restored keratinocyte membrane integrity (Supplemental Figure 5B). Similar to the responses of NHEKs to *S*. *hominis*, genes associated with lipid synthesis were greatly induced by CM from *Sa*Δ*agr* compared with WT *S*. *aureus* CM and the control (Figure 7B). Scratch-induced injury of NHEKs followed by *S*. *aureus* exposure led to reduced ELOVL4 protein expression, which was restored upon deletion of agr activity in *S*. *aureus* (Figure 7C). Furthermore, immunofluorescence staining of the murine wounds inoculated with *S*. *aureus*, *Sa*Δ*agr*, or vehicle control demonstrated increased ELOVL4 protein in the neoepithelium of the control wound and throughout the epithelium of the *Sa*Δ*agr* wound (Figure 7D). The absence of *S*. *aureus* agr products also restored NHEK proliferation (Figure 7E), which was supported by observations of increased expression of *KI67* (Figure 7F) and restoration of cell cycle genes *FOS*, *JUN*, and *JUNB* (Figure 7G) to levels at or above the control. Together, these data showed that *S*. *aureus* products under the control of the agr system impaired keratinocyte functions required for wound healing, including keratinocyte lipid metabolism and cell cycling.

### Identification of factors controlled by the agr that inhibit wound repair.

**Figure 8:**
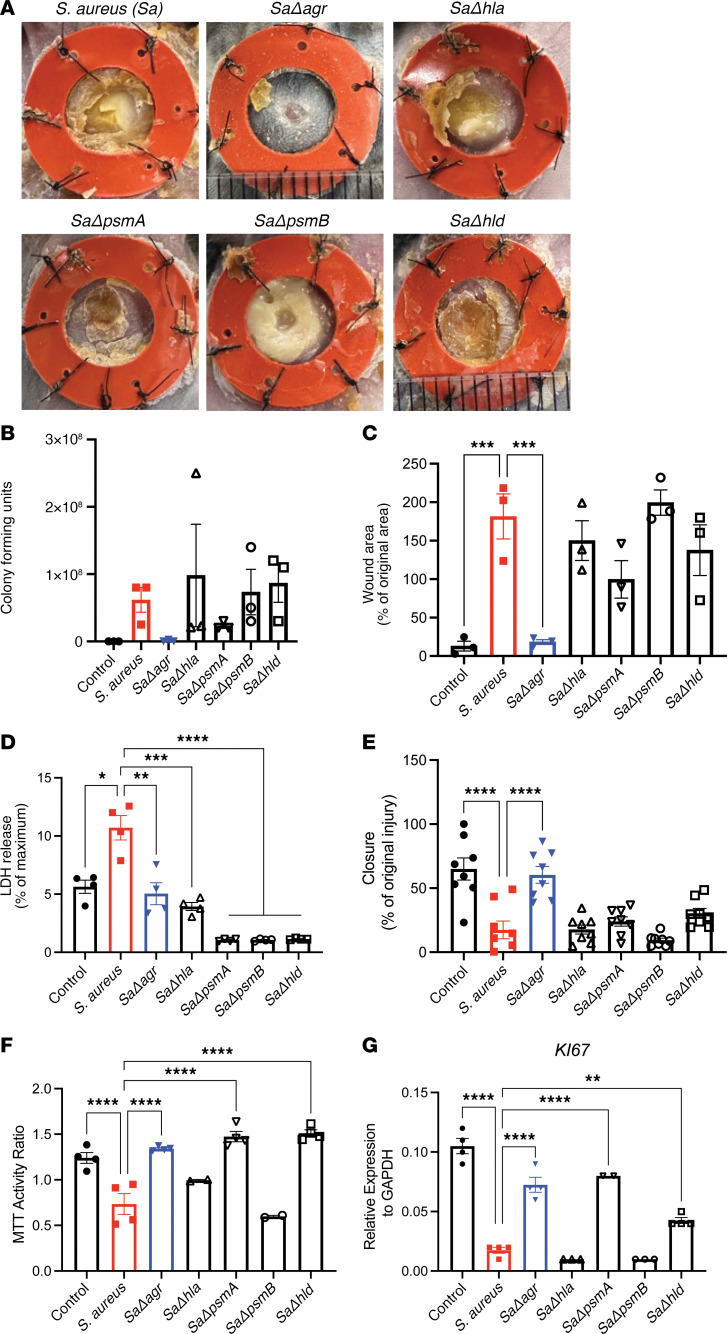
Differential effects on wound healing and gene expression by specific products of the *S*. *aureus* agr system. (**A**) Representative images of murine wounds 7 days after infection with indicated *S*. *aureus* mutants. (**B**) Bacteria recovered from day 7 murine wounds infected with *S*. *aureus* mutants. (**C**) Quantification of closure on day 7 of murine wounds infected with *S*. *aureus* mutants. (**D**) LDH release from scratch-wounded primary NHEKs 24 hours after treatment with CM of *S*. *aureus* mutants (3 × 10^7^ CFU/mL equivalent). (**E**) Scratch-wounded NHEK migration after 24-hour treatment with CM of *S*. *aureus* mutants (3 × 10^7^ CFU/mL equivalent). (**F**) Proliferation measured with MTT assay of NHEKs after 24-hour treatment with CM of *S*. *aureus* mutants (3 × 10^7^ CFU/mL equivalent). (**G**) *KI67* gene expression in proliferating NHEKs treated for 24 hours with CM of *S*. *aureus* mutants (3 × 10^7^ CFU/mL equivalent). One-way ANOVA followed by Bonferroni’s multiple-comparison adjustment for more than 2 groups (**B**–**G**). Experiments were performed at least twice. Data represent mean ± SEM. *P < 0.05, **P < 0.01, ***P < 0.001, ****P < 0.0001.

**Figure 9:**
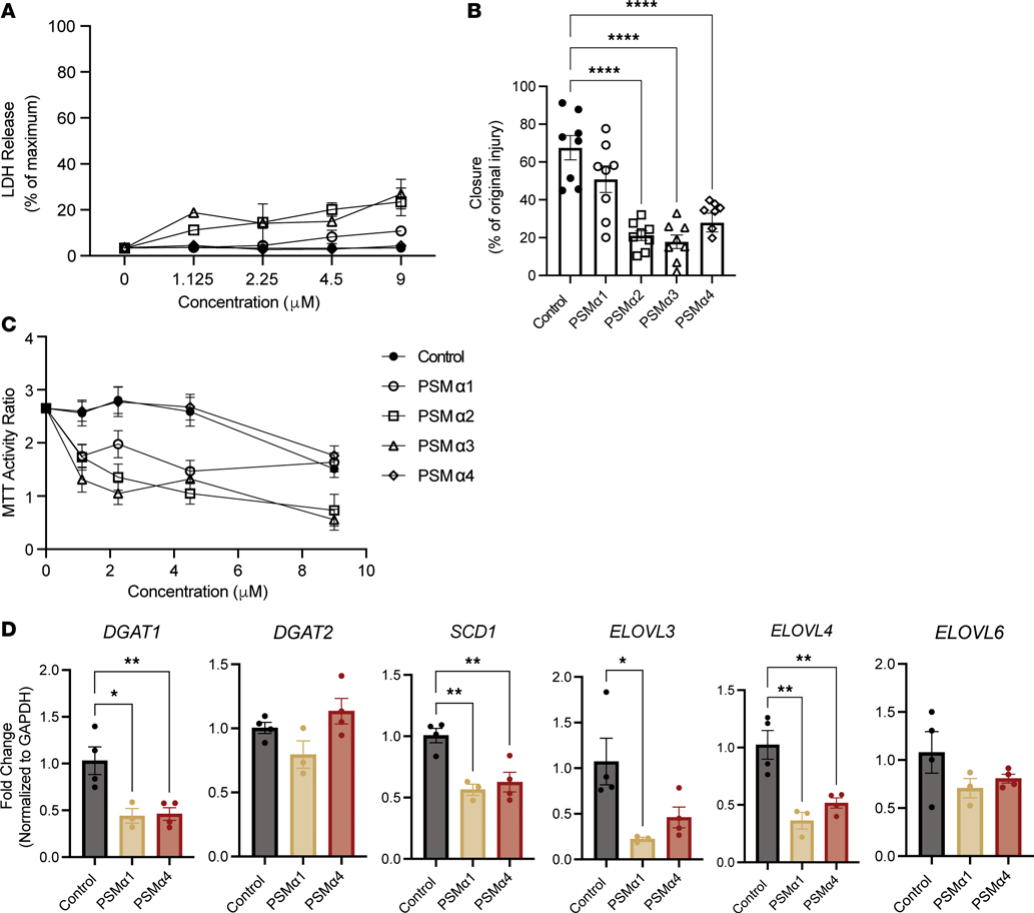
Each *S*. *aureus* PSMα peptide affects keratinocyte function distinctly. (**A**) LDH release as a percentage of maximum release of scratch-wounded NHEKs coincubated with various concentrations of synthetic PSMα1, PSMα2, PSMα3, or PSMα4 peptide or vehicle treatment (0.1% DMSO) for 24 hours. (**B**) Quantification of scratch-wounded NHEK migration treated for 24 hours with synthetic PSMα1, PSMα2, PSMα3, or PSMα4 peptide (2.25 μM) compared with vehicle treatment (0.1% DMSO). (**C**) Quantification of NHEK proliferation after 24 hours treatment with increasing doses of synthetic PSMα1, PSMα2, PSMα3, or PSMα4 peptide compared with vehicle treatment (0.1% DMSO). (**D**) qPCR quantification of lipid metabolic/synthesis enzyme gene expression in scratch-wounded NHEKs treated for 24 hours with synthetic PSMα1, PSMα2, PSMα3, or PSMα4 peptide (2.25 μM) compared with vehicle treatment (0.1% DMSO). One-way ANOVA followed by Bonferroni’s multiple-comparison adjustment for more than 2 groups (**A**–**D**). Experiments were performed at least twice. Data represent mean ± SEM. *P < 0.05, **P < 0.01, ****P < 0.0001.

Having determined that the *S*. *aureus* agr system is necessary to inhibit wound healing, we next attempted to define specific factors controlled by agr that are responsible for the effects observed on keratinocytes. Given that the agr system regulates multiple genes in *S*. *aureus*, we focused on the action of toxins and phenol-soluble modulins (PSMs) based on previous studies finding detrimental action of these products on intact skin (22). In mouse wounds, deletion of the *psmα* operon from *S*. *aureus* resulted in the most notable effect, with reduced CFUs and partial restoration of wound closure at day 7 after wounding compared with the *SaΔagr* mutant (Figure 8, A–C). Individual deletion of *hla*, *psmA*, *psmB*, or *hld* eliminated the capacity of *S*. *aureus* to cause LDH release from cultured NHEKs (Figure 8D), and loss of *hla* and *hld* ameliorated cell permeability observed with WT *S*. *aureus* (Supplemental Figure 6A). Although no individual deletion mutant restored NHEK migration to control or *SaΔagr* (Figure 8E), deletion of PSMαs or δ-toxin restored cell proliferation (Figure 8, F and G). The deletion of the *psma* operon also induced the expression of several lipid biosynthesis genes in NHEKs that were suppressed by WT *S*. *aureus* (Supplemental Figure 6B). Finally, treatment of NHEKs with 4 synthetic PSMα peptides showed differential effects of the products of the *psma* operon on keratinocyte permeability, migration, proliferation, and lipid biosynthesis. Each PSMα peptide affected NHEK transcription and function distinctively, with PSMα2 and α3 being the most cytotoxic and PSMα1 and α4 having opposite effects on function but similar effects on lipid biosynthesis gene expression (Figure 9, A–D). Collectively, these results suggest that targeting an individual PSM or toxin will not be sufficient to restore healing but rather, due to the distinct effects of specific *S*. *aureus* agr system products on keratinocytes, complete blockade of the agr system is required.

## Discussion

Our study aimed to better understand how *S*. *aureus* delays wound repair. By leveraging different RNA-Seq approaches, mouse and human model systems, and targeted bacterial mutagenesis, we sought to gain a deeper understanding of the host-pathogen interactions in the wound healing process that are critical for the delay of healing. Our findings revealed that *S*. *aureus* uniquely impaired healing compared with the human skin commensal *S*. *hominis*. The deleterious effect of infection with *S*. *aureus* on wound healing was density-dependent and did not occur in the absence of a functional agr quorum-sensing system. We further showed that *S*. *aureus* suppressed epidermal gene expression related to lipid metabolism in keratinocytes and that deletion of the agr quorum-sensing system in *S*. *aureus* restored both wound healing and lipid metabolism. We conclude that inhibition of the agr system of *S*. *aureus* presents a promising nonantibiotic therapeutic approach for improving wound repair and that keratinocyte lipid metabolism is a previously unappreciated target of *S*. *aureus*.

There are contradictory reports regarding the role of bacteria on skin repair that can be resolved by comparison of methodologies and consideration of the results of this current study. Wounds are not sterile but represent an environment where the physical barrier of the epidermis no longer inhibits commensal microbes. An example of a beneficial bacteria in diabetic wound repair is *Alcaligenes faecalis*, which was found to correct the aberrant overexpression of matrix metalloproteinases (12). Pathogens such as *S*. *aureus* can exploit this opportunity to cause infection despite rapid activation of host defense events, such as an alkaline pH shift, release of antimicrobial peptides from platelets, increased expression of antimicrobial peptides from keratinocytes, and recruitment of neutrophils (23–25). A critical density of bacteria above 10^5^ CFU/gm of tissue is used clinically to define conditions that result in delayed repair (26). Our observations are consistent with this threshold for *S*. *aureus* but not *S*. *hominis*.

Current approaches to defining the microbiota of chronic wounds and potential mechanisms of action involved in delayed repair have progressed considerably by understanding the roles of different species in this system (6). In acute wounds, decreasing microbial load delayed the expression of important wound defense factors such as CXCL10, while the addition of *S*. *epidermidis* to a mouse wound promoted wound closure (16). Indeed, we observed in vitro that exposure to *S*. *hominis* products promoted the expression of several lipid metabolic genes above that seen in sterile culture. Although *S*. *hominis* did not change wound repair compared with vehicle controls, there may be beneficial effects on a sterile wound. However, commensals such as *S*. *epidermidis* are also potential pathogens, have toxic agr products, and can delay barrier repair under different conditions (27). The work shown here demonstrates how keratinocytes are uniquely sensitive to different bacterial products. The overall host response will be dictated by both the concentration of these products and their combination. Thus, it is likely that acute wound repair may benefit from low levels of some microbes, but conclusions regarding which bacteria are beneficial or detrimental cannot be made based on species alone. The function of bacteria in the wound must be considered based on the identity of the strain, gene products, and dose.

Previous reports of DFUs demonstrate the complexity of multiple mechanisms, cell types, and functions that may contribute to the overall inhibition of wound healing in DFUs. For example, single-cell transcriptomic analysis of nonhealing DFUs focused primarily on differences in macrophage and fibroblast function in this condition (28). Other global transcriptomic studies of DFUs revealed poor control of the inflammatory response and decreased recruitment of neutrophils and macrophages, suggesting that the qualitative inflammatory response in DFUs is below the necessary threshold generated in acute human wounds (29). Further analyses of keratinocytes in DFUs show inhibition of their migration, in part by induced expression of miR193b-3p (30). Spatial transcriptomic results of this study focused attention on the early response of keratinocytes in the wound epithelium and thus identified the association with fatty acid metabolism and quorum sensing.

The clinical relevance of observations in mice and on human keratinocytes in vitro is supported by similar expression patterns for genes involved in fatty acid synthesis that were observed in human wounds infected by *S*. *aureus* and human skin that was infected ex situ. An exception is ELOVL3, which was found to be induced in infected DFUs but suppressed in infected keratinocytes and mouse wounds. This discrepancy is not surprising given the multiple different roles of ELOVL3. This gene has been reported to be suppressed in macrophages stimulated by lactic acid and negatively correlated with lactate levels in patients with sepsis (31) and in skin lesions from psoriasis and atopic dermatitis (32, 33). On the other hand, ELOVL3 was induced in mice that showed severe epidermal barrier impairment following a dominant-negative mutation of LEF1 (34) and was induced by norepinephrine and PPAR-agonists in brown adipocytes (35). Furthermore, ELOVL3 is found to be tightly regulated by the circadian clock (35–37), which can also explain the variability detected in expression levels. Taken together, ELOVL3 differential expression may be unique to human skin in this context and requires further exploration.

A limitation of the work presented here is the choice of *S*. *aureus* and *S*. *hominis* for study. The *S*. *aureus* strain USA300 LAC used here is a highly virulent strain of MRSA. Responses may differ to other *S*. *aureus* strains and will likely also be different from other pathogens that delay wound healing. *S*. *hominis* strain A9 is a beneficial isolate from normal human skin (38) and was used here in addition to *S*. *hominis* C2, but other strains may have different effects. Other species of coagulase-negative Staphylococci such as *S*. *epidermidis* can be pathogens and may result in different outcomes at the density tested, or perhaps other densities. Our observations in culture suggest *S*. *aureus* agr products are critical to target keratinocytes. However, other actions may occur in vivo since the *Sa*Δ*agr* mutant, as well as commensal *S*. *hominis*, were eliminated from the wound more quickly than WT *S*. *aureus*. Long-chain fatty acids are known to have antimicrobial functions on the skin, and *S*. *aureus*–derived lipase Lip2 was identified as a resistance factor against antimicrobial fatty acids (39). It is therefore possible that other factors, present when bacteria are at high density later in the course of healing, also contribute to delayed repair through modifying the host lipid environment or exacerbating inflammation. For example, a recent study of *S*. *aureus* lipase (gehB) under ArlRS 2-component system delays healing in a TLR-2–dependent manner (40). *Geh* is under agr control as well (41), and we have previously shown that it and other lipases are required for invasion of *S*. *aureus* into the hair follicle (42). Although we did not specifically test for lipases, future studies may address them as potential agr-regulated targets. Additionally, injury alone increases glycolysis of keratinocytes in the migrating front (21). Metabolizing *Staphylococcus* is capable of glycolysis, therefore taking glucose out of the microenvironment, although keratinocyte glycolysis and oxidative phosphorylation is not dependent on agr toxin expression (43). In addition to this, metabolizing *S*. *aureus* induces HIF1α production and accumulation in keratinocytes, which correlates to increased IL-1β secretion (43). For a complete understanding of the wound lipid metabolic microenvironment, both host and microbe contributions must be taken into account.

To our knowledge, the reduction of fatty acid synthesis by *S*. *aureus* during the early stages of the wound repair process has not been reported and requires further study. In our observations of cell responses to the mutation of the *psma* operon, we found that this deletion resulted in even higher expression of *DGAT1*, *SCD*, and *ELOVL6* than the complete agr mutation. Similarly, the addition of PSMα1 and PSMα4 synthetic peptides suppressed the expression of these enzymes. Thus, the PSMα family of peptides, while not solely responsible for delayed wound healing, may be particularly important agr products of the *S*. *aureus* quorum-sensing system. Whether this is an indirect result of keratinocytes shifting cellular resources from repair to host defense, or a direct impact of the *S*. *aureus* products themselves, or possibly the involvement of neighboring cells such as structural cells, requires further elucidation. Recent work from the Plikus lab has provided foundational work for fibroblast heterogeneity dynamics during wound healing (44). However, samples were not infected, and so further studies will be required to reveal the impact of bacteria on these fibroblast populations.

Overall, our results clearly identify the *S*. *aureus* agr system as a critical mechanism by which this important pathogen delays wound repair. Our findings that the *Sa*Δ*agr* mutant was rapidly eliminated from wounds after initial infection suggest targeting the agr system, or critical virulence products under its control, can be an alternative therapeutic approach to enable normal wound healing without the use of antibiotics. With the high prevalence of antibiotic resistance in *S*. *aureus* and the need to decrease the use of antibiotics to slow further development of this major health care problem, inhibition of the agr system offers a preventative approach that could limit the use of antibiotics. These findings further demonstrate the importance of understanding how microbes exert their pathogenic effect and provide a vital opportunity to gain deeper insights into the essential processes of wound repair.

## Methods

### Sex as a biological variable.

This study examined wounds in both male and female mice and tissue obtained from both men and women. Similar findings were observed for both sexes.

### Study design.

This study was designed to compare commensal and pathogenic bacterial species of *Staphylococcus* on murine skin wound repair, in both males and females, and reveal host-pathogen interactions that delay repair. Hypotheses based on discoveries made in vitro were tested on male and female patient samples collected at the University of Miami Hospital under the protocols approved by the IRB (protocols 20140473, 20090709, 20150222) and human wounds ex vivo. Murine wound closure and reepithelization were evaluated with planimetry and histology image analysis. Bacterial burden in murine wounds was quantified by live cultures of wound swabs. The wound tissue transcriptome was evaluated with spatial and single-cell RNA-Seq as well as validation with RT-qPCR. Direct impacts of bacterial products on keratinocyte migration and proliferation were evaluated in vitro. To further investigate specific *S*. *aureus* products responsible for delayed repair, genetic mutants of *S*. *aureus* were utilized for all models tested. No power analysis was performed to predetermine sample size. All experiments were repeated at least twice.

### Animals and animal care.

WT mice (female and male C57BL/6 mice, 000664, 12–16 weeks old) were obtained from The Jackson Laboratory. All animal experiments were approved by the University of California San Diego IACUC (protocol S09074). For all animal studies, animals were randomly selected without formal prerandomization, and quantitative measurements were done without the opportunity for bias.

### Bacterial strains.

USA300 is a predominant community-associated MRSA strain. The following MRSA strains were provided in-house: USA300 LAC WT (AH1263), USA300 LAC agrI P3-YFP (cmR) (AH1677) (45), USA300 LAC Δ*agr* (AH1292) (46), and USA300 Δ*hla* (AH1589). The following MRSA strains were provided by Michael Otto (Pathogen Molecular Genetics Section, NIAID, NIH, Bethesda, Maryland, USA): USA300 LAC Δ*psmA*, USA300 LAC Δ*psmB*, and USA300 LAC Δ*hld*. The following *S*. *hominis* strains were used and provided in-house: ShA9 and ShC2. All *Staphylococcus aureus* and *S*. *hominis* strains were grown overnight (18 hours) to stationary phase in 3% tryptic soy broth (TSB) at 300 rpm in a 37°C incubator unless stated otherwise. All staphylococci indicated were grown approximately to an OD600 nm reading of 10 or 3 × 10^9^ CFU/mL. For the treatment of bacterial supernatant on primary neonatal human epidermal keratinocytes, bacteria cultured overnight were pelleted (5 minutes, 0.4 *g* rpm, room temperature) and the supernatant was filter-sterilized (0.22 μm) prior to addition to cells.

### Mouse model of wound and infection.

Skin wound experiments were adapted from a splinted full-thickness wound model described previously (47). Briefly, 3 days before wounding, the dorsal skin of mice was shaved and then depilated (Nair, Church & Dwight). On day 0, mice were anesthetized with 2.5% isoflurane. After 2 betadine and alcohol washes of the skin, 1 sterile silicon splint (10 mm inner diameter, 16 mm outer diameter, 1.6 mm thick) was glued (Superglue, 3M) and secured with 6 interrupted sutures (6-0 Ethilon, 697G). Wounds were created in telogen hair-bearing skin, evidenced by the absence of visible dark follicles. One 4 mm full-thickness circular wound was created with a biopsy punch (Miltex Instruments, Integra Lifesciences) in the center of the splint. Wounds were infected with *S*. *aureus* strains listed in 10 μL of gel. The back skin including splint, wound, and skin surrounding splint was sealed with a transparent, semipermeable dressing (Tegaderm, 3M). The mice were housed singly after wounding, checked daily, and wounds were imaged as indicated.

### Murine wound model analysis.

For in vivo live bacterial imaging, mice were imaged under isoflurane inhalation anesthesia (2%). Photons emitted from luminescent bacteria were collected during a 1-second exposure using the Spectrum IVIS Imaging System and live image software (PerkinElmer). Bioluminescent or fluorescent image data were presented on a pseudocolor scale overlaid onto a gray-scale photographic image. Using the live imaging software (PerkinElmer) image analysis tools, circular analysis windows (of uniform area) were overlaid onto regions of interest, and the emitted photons (p/s/cm^2^/sr) were measured. For topical infection, live bacteria were applied in 10 μL of gel, with 10 μL of sterile gel used as a control. Mice were euthanized on days 1, 3, or 7 and tissues were collected for analysis as detailed below.

To count CFUs, vortexed swabs were serially diluted, plated onto tryptic soy agar, and enumerated after 18 hours to quantify the CFU per wound.

For histology and spatial sequencing, a 3 cm × 3 cm square of skin around the topical infection site or the wound infection was carefully dissected out, harvested, and fixed in 4% PFA for 24 hours at 4°C. Skin was washed 3 times with PBS, and then stored in 70% isopropanol until processed for paraffin embedding, sectioning, and histology.

For immunofluorescence, antigen retrieval of FFPE sections was performed using Target Retrieval Solution (Dako, S2369) per the manufacturer’s recommendations. Sections were blocked with serum from secondary antibody host, stained with primary antibodies overnight at 4°C, secondary antibodies for 1 hour at room temperature, and nuclei were counterstained with DAPI. Epifluorescence images were taken using an EVOS5000. Brightness and contrast were adjusted slightly using ImageJ (NIH) and applied equally across samples. The primary antibody was ELOVL4 (Proteintech, 55023-1-AP, 1:500). The secondary antibody was Cy3 donkey anti-rabbit IgG (BioLegend, 406402, 1:500).

For RT-qPCR, biopsy punch was used to collect skin surrounding the infected wound (6–8 mm). Skin was stored in RNALater solution at 4°C for 24 hours for RNA extraction. Skin biopsies were homogenized in 1 mL lysis buffer with 2 mm zirconia beads in a mini-bead beater 16 (BioSpec), and homogenate was processed using the PureLink RNA extraction kit as described below.

### Human DFU tissue samples.

Full-thickness skin from the wound edge of DFUs was obtained from consenting patients at the University of Miami Hospital under the protocols approved by the IRB (protocols 20140473, 20090709, 20150222) as previously described (4, 5, 30). Patients were treated with standard of care, debridement and offloading, and wound size measurements were performed weekly to determine healing outcomes and clinical symptoms of infection (48–50). Tissue samples were fixed in formalin for paraffin embedding, and histology quality control was performed as described in Stojadinovic et al. (51).

### DNA and RNA extraction from FFPE DFU samples.

Colonized (*n* = 5), infected (*n* = 5), healing (*n* = 4), and nonhealing (*n* = 4) DFU FFPE specimens were used for RNA and DNA extraction. Two 10 μm sections were collected from each specimen. DNA and RNA were isolated following the manufacturer’s instructions (Zymo Research, Quick DNA/RNA FFPE kit).

### qPCR of FFPE DFU samples.

Total RNA from control and infected ex vivo wounds was extracted using the Direct-zol RNA miniprep kit (Zymo Research). cDNA was synthesized with qScript cDNA Synthesis kit (QIAGEN). Further, RNA or DNA isolated from FFPE were preamplified using SSoAdvanced PreAmp Supermix kit (BioRad) with premixed primers according to the manufacturer’s protocol. 16S, (F 5′-AGAGTTTGATCCTGGCTCAG-3′, R 5′-GCTGCCTCCCGTAGGAGT-3′), *S*. *aureus* NucA (F 5′GTTGTAGTTTCAAGTCTAAGTAGCTC-3′, R 5′-AACCGTATCACCATCAATC-3′), and ARPC2 (F 5′-TCCGGGACTACCTGCACTAC-3′, R 5′-GGTTCAGCACCTTGAGGAAG-3′) were used as reference genes. All qPCR reactions were done in duplicate using the PerfeCTa SYBR Green SuperMix (QuantaBio), and the relative gene expression was calculated with the 2^–ΔΔCT^ method.

### Primary NHEKs.

Primary NHEKs (ATCC, PCS-200-010) were cultured in EpiLife medium containing 60 μM CaCl_2_ (Gibco, MEPI500CA) supplemented with Human Keratinocyte Growth Supplement (Gibco, S0015) and antibiotic-antimycotic (penicillin 100 U/mL, streptomycin 100 U/mL, and amphotericin B 250 ng/mL; Gibco, 15240062) at 37°C, 5% CO_2_. For experiments, NHEKs were only used for experiments between passages 3 and 5. For bacterial supernatant treatments, undifferentiated NHEKs were treated with sterile-filtered bacterial supernatant at 3% by volume to EpiLife medium for up to 24 hours. This volume was selected to ensure secreted product treatment while limiting cytotoxicity of bacteria culture media in keratinocyte media. Similarly, for synthetic PSM treatments, the peptides were added to the NHEKs for up to 24 hours in a final volume of 0.1% DMSO and 2.25 mM concentration. Migration and proliferation studies were conducted as stated below.

### Cell proliferation.

To measure cell viability and proliferation, HaCaT cells or NHEKs were seeded at 2 × 10^4^ cells/well in 12-well plates in DMEM supplemented with 5% FBS or complete EpiLife medium, respectively. After a 24-hour incubation, sterile-filtered CM was added to the wells. Cultures were treated with CM, TSB (negative control), or media only (untreated control). For the positive control, designated wells were treated with lysis buffer for 3 hours according to the manufacturer’s instructions. The colorimetric MTT assay (Abcam, ab211091) for measuring cell viability and proliferation was performed after 24 hours. Absorbance was read on a microplate reader (Molecular Devices SpectraMax iD3) at 570 nm.

### Scratch wound assay.

For scratch wound assays, HaCaT cells in DMEM containing 5% FBS or NHEKs in EpiLife media supplemented with human keratinocyte growth supplement (HKGS) were grown to 100% confluence in 24-well plates. To inhibit proliferation, cells were treated with 10 μg/mL mitomycin for 1 hour before scratching and washed with media. Scratches were performed with a 200 μL pipette tip, and then washed 2 times with media. Cells were incubated with CM, left untreated (positive control), or treated with TSB (negative control). At least 8 scratched areas for each sample were marked and photographed immediately at 0 hour and at 24 hours with an inverted microscope. Migration was evaluated by measuring the defect width at 0 and 24 hours after scratching using ImageJ (NIH) software and calculating the averages and SEM of defect difference between time 0 and 24 hours for each treatment group.

### LDH assay.

The LDH cytotoxicity assay was performed according to the manufacturer’s instructions (CyQUANT LDH cytotoxicity assay, Invitrogen, C20300). Cell culture supernatants from keratinocytes were used fresh for the LDH assay after experimental treatments.

### RNA isolation and RT-qPCR of primary keratinocytes.

RNA was isolated using the PureLink RNA isolation kit according to the manufacturer’s instructions (Invitrogen, 12183018A). For NHEKs, 250 μL of RNA lysis buffer was added directly to cells prior to addition of 250 μL of 70% EtOH and column-based isolation of RNA. For mouse tissue, full-thickness skin was subjected to bead-beating in 750 μL of RNA lysis buffer (3 × 30 seconds with 5 minutes on ice in between, 2.0 mm zirconia bead). Tissue was then centrifuged (10 minutes, 4 *g*, 4°C), followed by the addition of 350 μL of clear lysate to 70% EtOH and column-based isolation of RNA. After RNA isolation, samples were quantified with a Nanodrop (Thermo Fisher Scientific, ND-2000), and 750 ng of human or mouse RNA was reverse-transcribed using the iScript cDNA synthesis kit (Bio-Rad, 1708890). RT-qPCR reactions were run on a CFX96 Real-Time Detection System (Bio-Rad). For both human and mouse cDNA, 2x SYBR Green qPCR Master Mix was used along with specific primers.

### Protein extraction and immunoblot analysis of primary keratinocytes.

Cultured NHEKs were lysed in radioimmunoprecipitation assay buffer (Cell Signaling Technology, 9806) supplemented with a complete proteinase and phosphatase inhibitor cocktail (Thermo Fisher Scientific, A32959). Protein concentrations were determined using a bicinchoninic acid protein assay kit (Thermo Fisher Scientific, A55864). Proteins were separated on a Novex Tricine protein gel (10% to 20%; Thermo Fisher Scientific, EC66252BOX). Then proteins were transferred onto a PVDF membrane using the Trans-Blot Turbo transfer pack (Bio-Rad, 1704156) and Trans-Blot Turbo transfer system (Bio-Rad, 1704150EDU). Membranes were blocked with Intercept (PBS) blocking buffer (LI-COR, 927-70001) for 2 hours at room temperature and subsequently incubated overnight at 4°C with primary antibodies against ELOVL4 (1:500; Proteintech, 55023-1-AP) or GAPDH (1:2,000; Cell Signaling Technology, 2118S). The following day, membranes were incubated with a secondary antibody (LI-COR, 926-68073) for 1 hour at room temperature. Protein bands were visualized using the Odyssey Classic Imager (LI-COR).

### Infection of human skin ex vivo wound model.

Healthy human skin samples were collected under the approved University of Miami Hospital IRB protocol (IRB 00007772) and used to generate acute wounds as previously described (4, 30, 52). Briefly, a 3 mm biopsy punch was used to create acute wounds in the epidermis. Skin discs (8 mm) with the 3 mm epidermal wound in the center were transferred to the air-liquid interface and maintained at the air-liquid interface at 37°C in a humidified atmosphere of 5% CO_2_. For wound infection, 10 μL of 10^8^ CFU/mL *S*. *aureus* USA300 was applied topically at the time of wounding. Infected and control uninfected wounds were processed at day 4 after wounding for RNA isolation and CFU quantification as described (30, 52).

### Synthetic S. aureus PSM preparation.

All synthetic PSMs were produced by LifeTein. Peptides were produced at 95% purity with N-terminal formylation. Peptides were resuspended in DMSO and concentrated by SpeedVac into 500 mg of powder stocks stored at –80°C and resuspended in DMSO to a stock concentration of 10 mM prior to further dilution in tissue culture growth medium for experiments. The following peptide sequences were generated: PSMα1:fMGIIAGIIKVIKSLIEQFTGK; PSMα2:fMGIIAGIIKFIKGLIEKFTGK; PSMα3:fMEFVAKLFKFFKDLLGKFLGNN; and PSMα4: fMAIVGTIIKIIKAIIDIFAK.

### Bulk RNA-Seq.

mRNA was extracted with the PureLink RNA isolation kit (Invitrogen, 12183018A) from NHEKs, followed by quality control and library preparation at the University of California San Diego Institute for Genomic Medicine Genominics Center. mRNA quality was assessed on an Agilent 2100 Bioanalyzer to identify samples with RNA integrity number 7 and greater, and an Illumina stranded mRNA prep kit was used. A NovaSeq 6000 S4 platform was used yielding an average of 25 million stranded 100-bp paired-end reads per sample. Libraries were constructed using TruSeq Stranded mRNA Library Prep kits (Illumina, 20020595) and run on a HiSeq 2500 instrument (Illumina). Raw data were analyzed using Partek Flow and Partek Genomics Suite software to determine transcript abundance and genes differentially expressed between samples. Gene Ontology analysis was performed using Metascape (http://metascape.org).

### scRNA-Seq.

Tissue samples from 3 mice in each group (control intact, *S*. *aureus* intact, control wound, *S*. *aureus* wound, *S*. *aureusΔagr* wound) were minced with a razor blade into 1 cm fragments, suspended in enzymatic digestion buffer containing collagenase and DNase I as previously described (53), incubated with frequent agitation at 37°C for 30 minutes, and triturated briefly with a 5 mL pipet. Cells in a single-cell suspension were then passed through a 100 μm mesh filter and loaded on the 10x Genomics Chromium system.

### Library construction protocol.

Single-cell suspensions were loaded onto the 10x Genomics Chromium Controller instrument to generate single-cell Gel Bead-in-Emulsion (GEMs). GEM-reverse transcriptomics and library construction were performed following the 10x Genomics protocol. Library fragment size distributions were determined using an Agilent Bioanalyzer High Sensitivity chip, and library DNA concentrations were determined using a Qubit 2.0 Fluorometer (Invitrogen). Libraries were sequenced using an Illumina HiSeq 4000.

### Data analysis.

For mouse skin, the 10x Genomics Cell Ranger version 7.0.1 and Space Ranger version 2.0.1 software pipeline with default parameters was used to perform sample demultiplexing, barcode processing, alignment to the mm10 reference genome, and single-cell gene counting. Data were further filtered, processed, and analyzed using the Seurat R toolkit version 4.0.6 (54, 55). Filtering of initial data involved selecting cells with more than 200 and less than 3,000 features and less than 15% mitochondrial genes. For single-cell sequencing, data were combined by IntegrateData(). The data were normalized using the SCTransform()function with parameters normalization.method = SCT. Prior to integrating the data, data features and anchors were identified with SelectIntegrationFeatures() and FindIntegrationAnchors(). Principal components were calculated from these variable genes using the function RunPCA(). Nonlinear dimensionality reduction and visualization was performed with UMAP (56) using the RunUMAP()function. Clusters were identified using the FindNeighbors()function using the significant PCs, and then the FindClusters() function with the parameter resolution = 0.5. Marker genes for clusters and between samples were determined using the FindAllMarkers()function with parameters min.pct = 0.25 and thresh.use = 0.25. Gene ontology analysis was performed on marker genes using the clusterProfiler R package with default parameters (57). The Seurat object was transformed into a SingleCellExperiment object and used for follow-on analysis. Marker genes were identified using the FindAllMarkers() function with parameters test.use = LR, latent.vars = Exp, min.pct = 0.25, and logfc.threshold = 0.4054651 (corresponding to 1.5 fold-change).

### Spatial transcriptomics.

Skin tissues from unwounded and wounded mice with and without infection were collected as described above. Fixed and washed tissues were processed and paraffin embedded, then sectioned to fit 11 mm^2^ oligo-barcoded capture areas on the Visium 10x Genomics slide. Before performing the complete protocol, Visium Spatial Tissue Optimization (10x Genomics) was performed according to the manufacturer’s instructions, and the fluorescent footprint was imaged using a Metafer Slide Scanning Platform (Metasystems). Nine minutes was selected as optimal permeabilization time. The experimental slide with colonic tissue was fixed and stained with H&E and imaged using Keyence BZX-700 Fluorescent Microscopy at 2× original magnification. Sequence libraries were then processed according to the manufacturer’s instructions (10x Genomics, Visium Spatial Transcriptomic).

For spatial sequencing, data were combined by merge(). Principal components were calculated from these variable genes using the function RunPCA(). Nonlinear dimensionality reduction and visualization was performed with UMAP (56) using the RunUMAP()function. The data were normalized using the SCTransform()function with parameters normalization.method = SCT. Clusters were identified using the FindNeighbors()function using the significant PCs and then the FindClusters() function with the parameter resolution = 0.5. Marker genes for clusters and between samples were determined using the FindAllMarkers()function with parameters min.pct = 0.25 and thresh.use = 0.25. Gene ontology analysis was performed on marker genes using the clusterProfiler R package with default parameters (57). The Seurat object was transformed into a SingleCellExperiment object and used for follow-on analysis. Marker genes were identified using the FindAllMarkers() function with parameters test.use = LR, latent.vars = Exp, min.pct = 0.25, and logfc.threshold = 0.4054651 (corresponding to 1.5 fold-change).

### IPA.

IPA (QIAGEN) was performed on differentially expressed genes with a *P* value of 0.05 or less and |fold-change| of 1.5 or greater, and Core Analysis was used to detect distinct and/or jointly regulated pathways and biological processes in cluster 11 and nonhealing DFUs datasets (GSE134431). The gene networks were built using the IPA builder tools, connecting and expanding the interactions between genes involved in chosen processes and functions.

### Statistics.

Mann-Whitney *U* test, 2-tailed Student’s *t* tests, and 1-way or 2-way ANOVA (parametric and nonparametric) were used for statistical analysis as indicated in the figure legends. The number of human skin specimens and independent ex vivo experiments are represented by *n* in the figure legends. All statistical analysis was performed using GraphPad Prism (version 8.0). All data are presented as mean ± SEM, and a *P* value of 0.05 or less was considered significant.

### Study approval.

DFU samples were obtained from consenting patients at the University of Miami Hospital under the protocols approved by the IRB (protocols 20140473, 20090709, 20150222). All animal experiments were approved by the University of California San Diego IACUC (protocol S09074).

### Data availability.

Values for data points in figures are reported in the Supporting Data Values file. IPA to detect distinct and/or jointly regulated pathways and biological processes in cluster 11 and nonhealing DFU datasets are available in NCBI’s Gene Expression Omnibus (GEO GSE134431). The GEO accession number for DFU single-cell RNA-Seq data used in deconvolution is GSE165816. Murine sequencing data has been deposited in GEO under accession numbers GSE134431 and GSE97617.

## Author contributions

MDB, IP, MTC, ARH, and RLG were responsible for conceptualization and methodology of the experiment, as well as funding acquisition. MDB, JM, NJ, TD, SA, EL, FL, HC, and ARM performed experiments and acquired and analyzed data. Data visualization was completed by MDB, JM, TD, and KJC. The project was supervised by RLG and administered by MDB and RLG, and the original draft was written by MDB and RLG. The manuscript was reviewed and edited by MDB, JM, IP, MTC, ARH, and RLG.

## Supplementary Material

Supplemental data

Supporting data values

## Version 1 10/15/2025

Electronic publication
