# Supplementary material for: Staphylococcus aureus accessory gene regulator quorum-sensing system inhibits keratinocyte lipid enzymes and delays wound repair
Source: J Clin Invest. 2025 Oct 15;135(20):e190411. doi: 10.1172/JCI190411 (PMC12520687; doi:10.1172/JCI190411)
Supplement: Supplemental data [file jci-135-190411-s010.pdf]

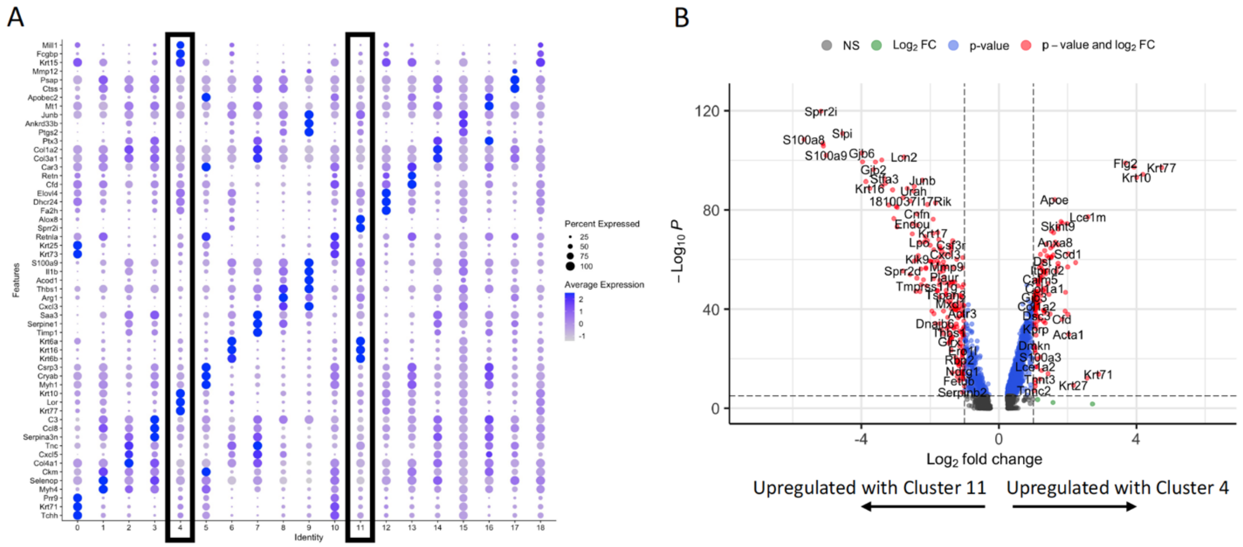

Supplemental Figure 1

(A) Top three overrepresented markers by cluster for Visium spatial RNAseq results with clusters 4 and 11 highlighted, (B) Differential gene expression comparison of Visium spatial RNAseq clusters 4 and 11.

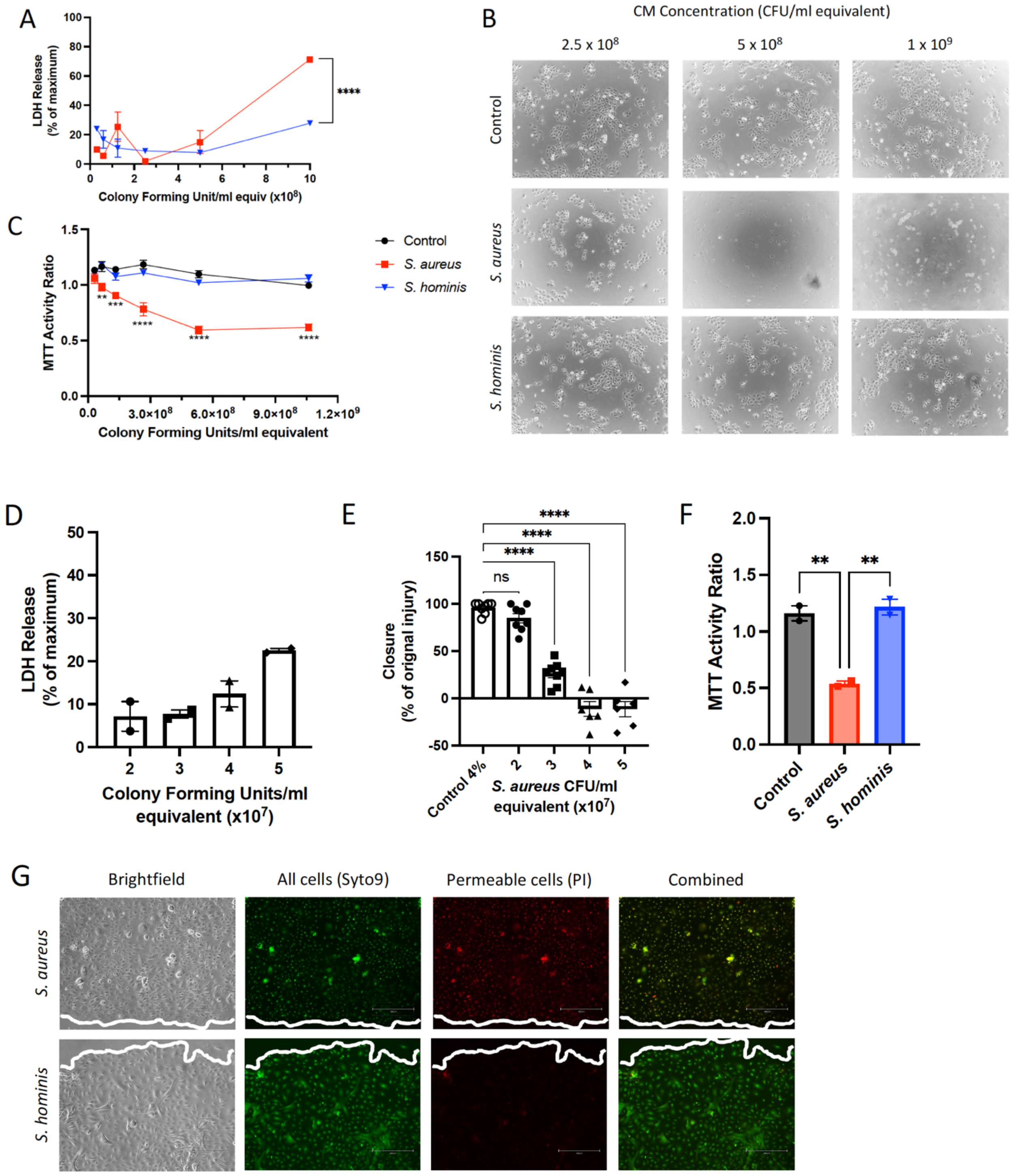

Supplemental Figure 2

(A) LDH release of HaCat keratinocytes treated with increasing doses of sterile conditioned media (CM) of either *S. aureus* or *S. hominis*, (B) Representative images of proliferating HaCat cells in the presence of CM from *S. aureus* or *S. hominis*, (C) Quantification of HaCat cell proliferation after treatment with increasing doses of CM from *S. aureus* or *S. hominis*, (D) LDH release of

NHEK treated with increasing concentrations of CM from *S. aureus*, (E) Quantification of wound closure *in vitro* NHEK after 24 hours of increasing concentrations of CM from *S. aureus* compared to 4% control, (F) Quantification of NHEK proliferation after CM from *S. aureus* or *S. hominis* (3x107 CFU/ml equivalent concentration) treatment, (G) Representative images of PI stained injured NHEK after 24 hour treatment of CM from *S. aureus* or *S. hominis*. *$P < 0.05$, **$P < 0.01$, ***$P < 0.001$, ****$P < 0.0001$, 1-way ANOVA followed by Bonferroni's multiple-comparison adjustment (A,C,D-F).

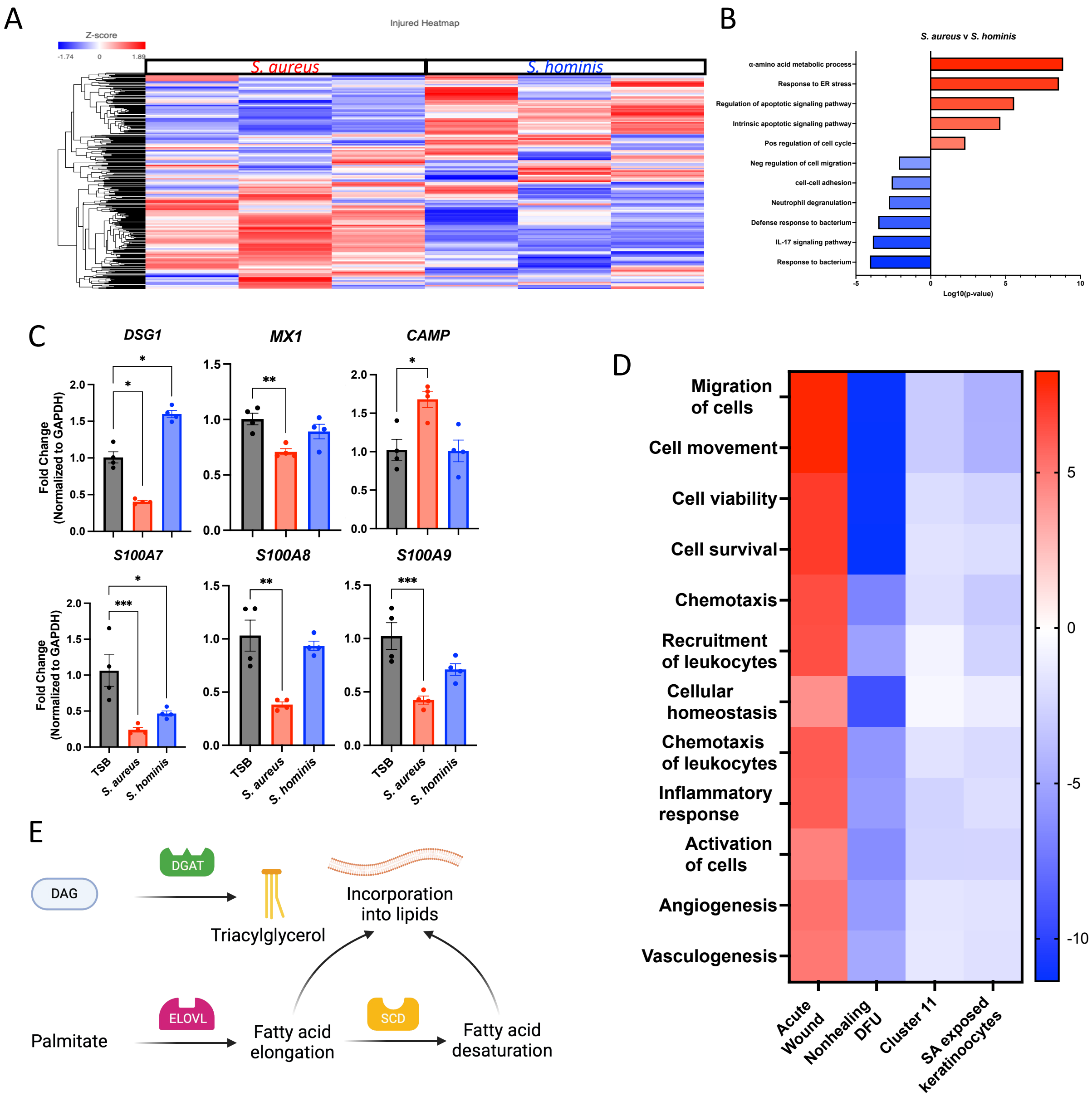

Supplemental Figure 3

(A) Heatmap of *S. aureus* versus *S. hominis* CM treated NHEKs after injury, (B) Top ten up and downregulated functional pathways of NHEKs after injury and 24 hour treatment with *S. aureus* versus *S. hominis* CM, (C) qPCR of cell-to-cell adhesion and defense genes expressed by injured NHEKs exposed to CM for 24 hrs, (D) Heatmap of IPA pathways comparing RNAseq data of human acute wounds and non-healing DFU, cluster 11 of murine wounds, and *S. aureus* exposed injured NHEKs, (E) Simplified schematic of lipid metabolic and synthesis enzymes. Diacylglycerol (DAG) is converted to triacylglycerol by diacylglycerol acyltransferase (DGAT). Palmitate is elongated by elongase enzymes (ELOVL) and desaturated by stearoyl-CoA desaturase (SCD) to form very long and desaturated fatty acids. *P < 0.05, **P < 0.01, ***P < 0.001, ****P < 0.0001, 1-way ANOVA followed by Bonferroni's multiple-comparison adjustment (C).

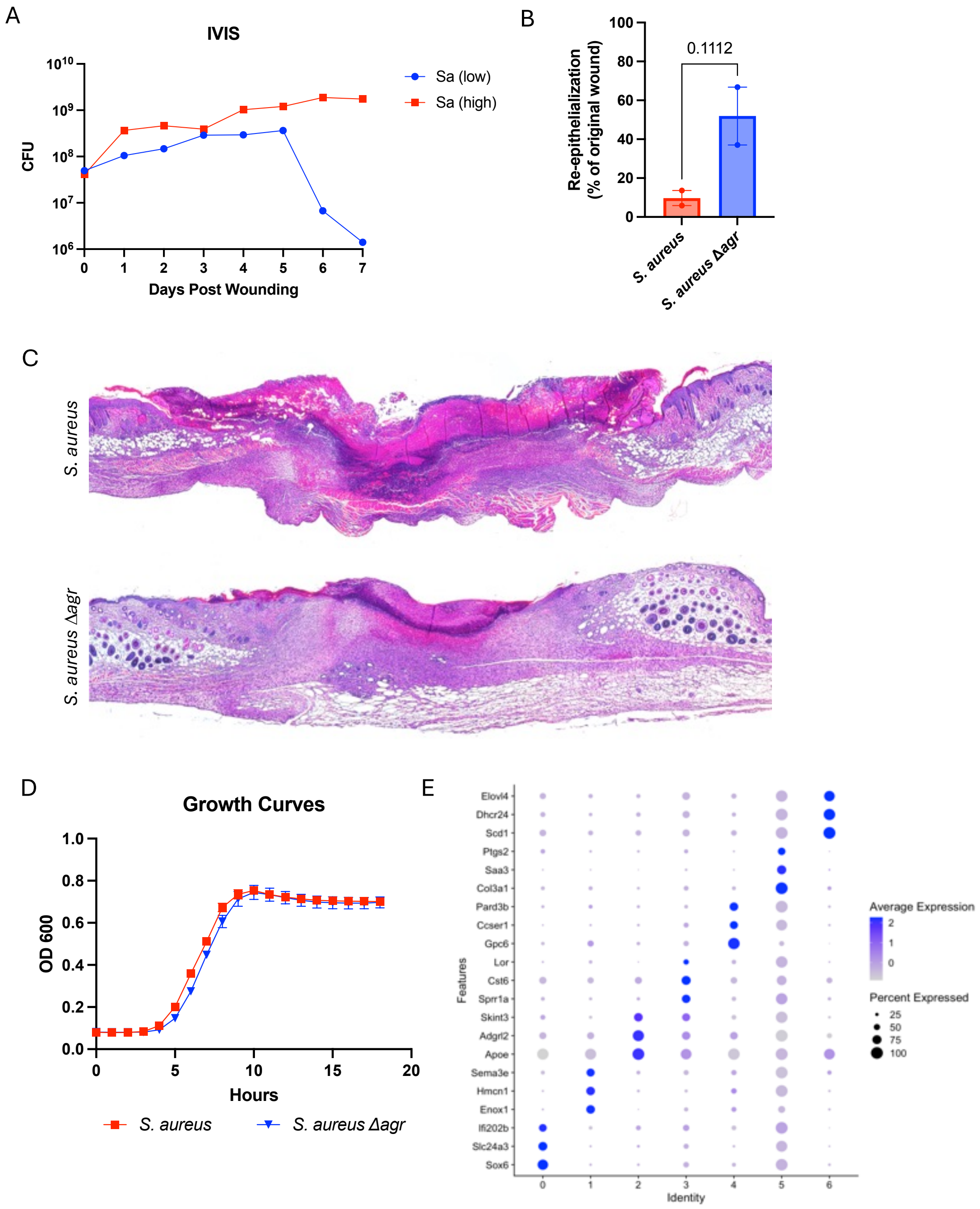

Supplemental Figure 4

(A) Representative bacterial burden kinetics measured by IVIS imaging of low v high *S. aureus* inoculation, (B) Quantification of re-epithelialization of *S. aureus* and *S. aureus*Δagr infected wounds on day 7 based on analysis of H&E histology images, (C) Representative H&E histology images of S. aureus and *S. aureus*Δagr infected wounds on day 7, (D) Bacterial growth curves for WT *S. aureus* parent strain and agr mutant cultured overnight in TSB media, (E) Top three overrepresented markers for each keratinocyte cluster. Student's t-test for comparison of two groups (B,D).

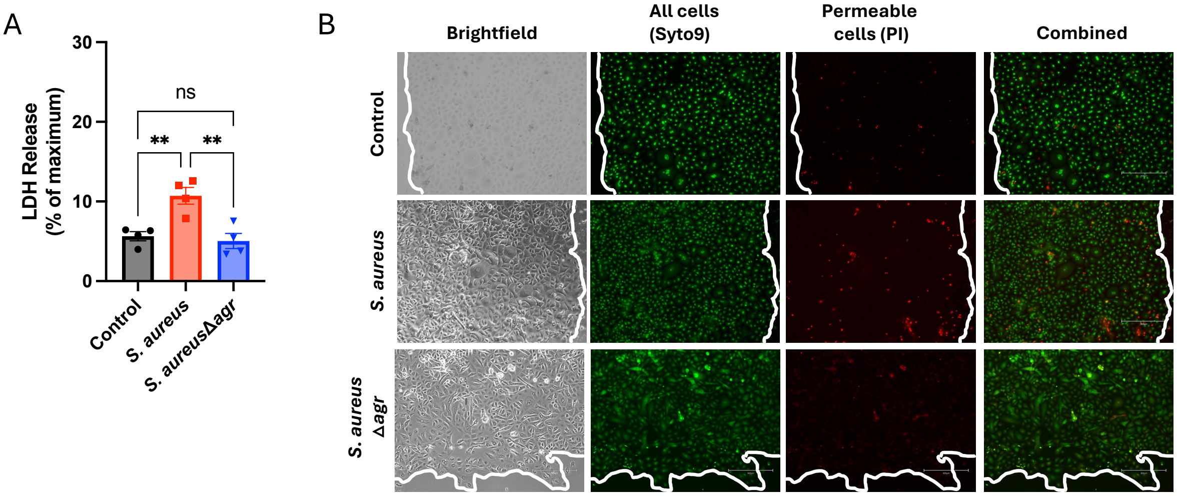

Supplemental Figure 5

(A) Quantification of LDH release from NHEK after 24-hour treatment with *S. aureus* CM, *S. aureus*∆agr CM (3x10^7 CFU/ml equivalent concentration), or media control, (B) Representative images of PI staining of NHEKs injured then treated with wild-type *S. aureus* or *S. aureus*∆agr CM or TSB control. *P < 0.05, **P < 0.01, ***P < 0.001, ****P < 0.0001, 1-way ANOVA followed by Bonferroni's multiple-comparison adjustment (A).

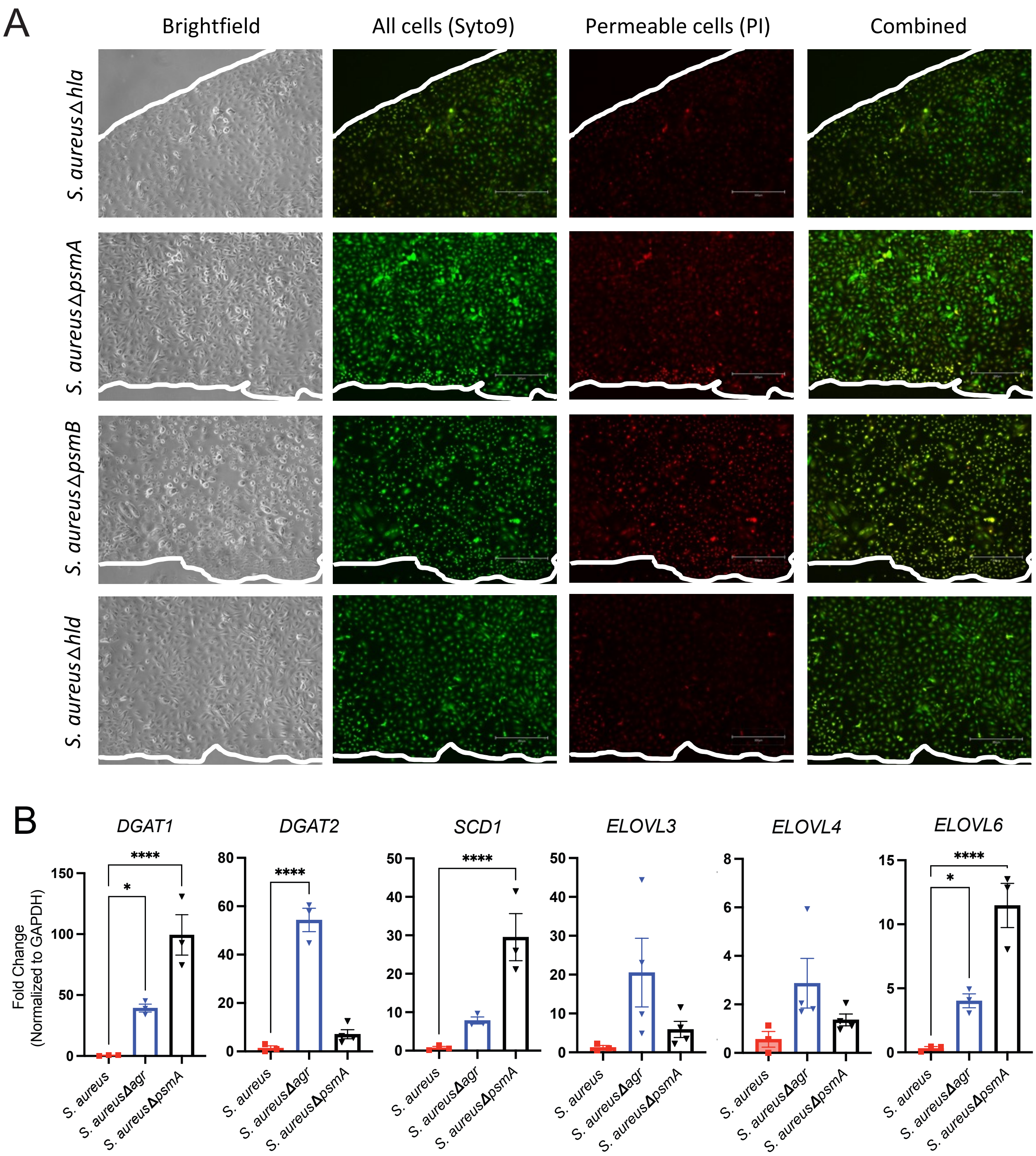

Supplementary Figure 6

(A) Representative images of Propidium iodide (PI) staining of NHEKs injured and then treated for 24 hours with the sterile conditioned media from the indicated mutants of *S. aureus*, (B) RT-qPCR quantification lipid metabolic/synthesis enzyme gene expression in scratch wounded NHEKs treated for 24 hours with *S. aureus* CM, *S. aureusΔagr* CM, or *S. aureusΔpsmA* CM ($3x10^7$ CFU/ml equivalent concentration). $^*P < 0.05$, $^{**}P < 0.01$, $^{***}P < 0.001$, $^{****}P < 0.0001$,

1-way ANOVA followed by Bonferroni's multiple-comparison adjustment for more than two groups (B).
